# A Computational Framework for Ultrastructural Mapping of Neural Circuitry

**DOI:** 10.1371/journal.pbio.1000074

**Published:** 2009-03-31

**Authors:** James R Anderson, Bryan W Jones, Jia-Hui Yang, Marguerite V Shaw, Carl B Watt, Pavel Koshevoy, Joel Spaltenstein, Elizabeth Jurrus, Kannan UV, Ross T Whitaker, David Mastronarde, Tolga Tasdizen, Robert E Marc

**Affiliations:** 1 Department Ophthalmology, Moran Eye Center, University of Utah, Salt Lake City, Utah, United States of America; 2 Sorenson Media, Salt Lake City, Utah, United States of America; 3 Scientific Computing and Imaging Institute, University of Utah, Salt Lake City, Utah, United States of America; 4 The Boulder Laboratory For 3-D Electron Microscopy of Cells, University of Colorado, Boulder, Colorado, United States of America; 5 Department Electrical and Computer Engineering, University of Utah, Salt Lake City, Utah, United States of America; University of Texas, United States of America

## Abstract

Circuitry mapping of metazoan neural systems is difficult because canonical neural regions (regions containing one or more copies of all components) are large, regional borders are uncertain, neuronal diversity is high, and potential network topologies so numerous that only anatomical ground truth can resolve them. Complete mapping of a specific network requires synaptic resolution, canonical region coverage, and robust neuronal classification. Though transmission electron microscopy (TEM) remains the optimal tool for network mapping, the process of building large serial section TEM (ssTEM) image volumes is rendered difficult by the need to precisely mosaic distorted image tiles and register distorted mosaics. Moreover, most molecular neuronal class markers are poorly compatible with optimal TEM imaging. Our objective was to build a complete framework for ultrastructural circuitry mapping. This framework combines strong TEM-compliant small molecule profiling with automated image tile mosaicking, automated slice-to-slice image registration, and gigabyte-scale image browsing for volume annotation. Specifically we show how ultrathin molecular profiling datasets and their resultant classification maps can be embedded into ssTEM datasets and how scripted acquisition tools (*SerialEM*), mosaicking and registration (*ir-tools*), and large slice viewers (*MosaicBuilder, Viking*) can be used to manage terabyte-scale volumes. These methods enable large-scale connectivity analyses of new and legacy data. In well-posed tasks (e.g., complete network mapping in retina), terabyte-scale image volumes that previously would require decades of assembly can now be completed in months. Perhaps more importantly, the fusion of molecular profiling, image acquisition by *SerialEM*, *ir-tools* volume assembly, and data viewers/annotators also allow ssTEM to be used as a prospective tool for discovery in nonneural systems and a practical screening methodology for neurogenetics. Finally, this framework provides a mechanism for parallelization of ssTEM imaging, volume assembly, and data analysis across an international user base, enhancing the productivity of a large cohort of electron microscopists.

## Author Summary

Building an accurate neural network diagram of the vertebrate nervous system is a major challenge in neuroscience. Diverse groups of neurons that function together form complex patterns of connections often spanning large regions of brain tissue, with uncertain borders. Although serial-section transmission electron microscopy remains the optimal tool for fine anatomical analyses, the time and cost of the undertaking has been prohibitive. We have assembled a complete framework for ultrastructural mapping using conventional transmission electron microscopy that tremendously accelerates image analysis. This framework combines small-molecule profiling to classify cells, automated image acquisition, automated mosaic formation, automated slice-to-slice image registration, and large-scale image browsing for volume annotation. Terabyte-scale image volumes requiring decades or more to assemble manually can now be automatically built in a few months. This makes serial-section transmission electron microscopy practical for high-resolution exploration of all complex tissue systems (neural or nonneural) as well as for ultrastructural screening of genetic models.

## Abstract 3

A framework for analysis of terabyte-scale serial-section transmission electron microscopic (ssTEM) datasets overcomes computational barriers and accelerates high-resolution tissue analysis, providing a practical way of mapping complex neural circuitry and an effective screening tool for neurogenetics.

## Introduction

Neural network reconstruction is a grand challenge in neuroscience and vision science in particular. Defining complete network (CN) maps or connectomes [1,2] for canonical regions of any metazoan neural assembly requires robust cataloguing of neuronal classes [3–7], mapping statistically distinct neuronal patterns [8–11], and tracing characteristic connections [12–14]. Moreover, anatomic methods for network analysis have not kept pace with the demands for phenotyping an immense and expanding library of genetic models of neurologic disorders in general [15] and retinal disorders in particular [16]. This is all the more distressing since, historically, anatomy has shown far more power to define neural network ground truth than either modeling or physiological strategies and, in practice, serial section transmission electron microscopy (ssTEM) has been the most powerful generator of validated existing network maps. The term “ground truth” emerged first in remote sensing and refers to specific ground information used to validate optical data captured from afar. In neuroscience, ground truth is the physical connectivity of identified neurons. Patterns of connectivity inferred from behavior, modeling, or physiology are thus subject to the test of anatomical ground truth. The need to expand ssTEM abilities beyond the purview of a limited number of specialized laboratories has never been more acute.

We have developed a complete suite of software tools and strategies that leverage existing ultrastructural resources (Table 1). Commercial [17] and academic [18,19] software solutions for small-scale, user-guided mosaicking and multimodal registration have long been available, but have not proven viable for large datasets or high throughput. By providing tools to precisely and automatically tile many images (≈1,000) into large mosaics, to precisely register serial mosaics (including multimodal frames), and to browse gigabyte image sets and terabyte volumes, we hope to enable expanded analysis of connectivity patterns in legacy as well as new image databases.

**Table 1:**
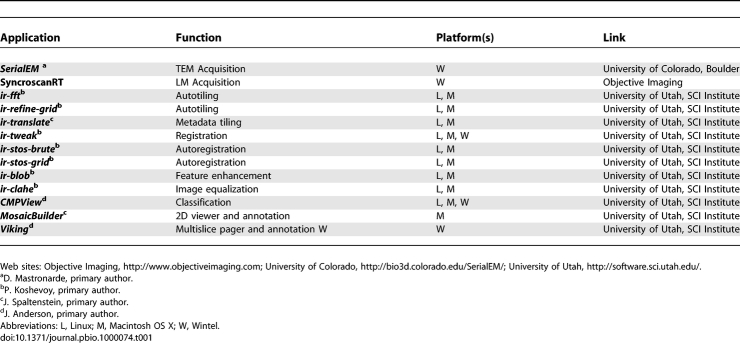
Software Tools and Sources

Why are such tools important? Simply, the unraveling of connective patterns in complex neural tissue and the characterization of deranged circuitry in disease states requires sampling scales that have been impractical. Some important neural reconstruction tasks are so large that they transcend investigator lifetimes using current resources [20]. The volume that must be constructed to approach sampling completeness in the inner plexiform layer of the mammalian retina is three orders of magnitude larger than most typical ssTEM volumes used in central nervous system studies [18,21]. Other programs are addressing these challenges by developing novel platforms to acquire pre-aligned serial electron microscope images [22–25]. However these platforms alone are not the sole nor optimal strategies for ssTEM volume assembly, as some new methods destroy samples, are limited in resolution and speed (Table 2), and most of the platforms are developmental or highly restricted in availability. Conversely, ssTEM has high resolution, tremendously flexibility in staining and immunocytochemical options, very fast acquisition times, and the potential for parallelization, by analogy with grid computing, via the subdivision of ssTEM samples into packets for parallel data acquisition. Data assembly could be readily done if tools to harmonize the effort were widely available. We present the essential software tools here, specifically those for assembling large-scale mosaics and achieving slice-to-slice image registration.

**Table 2:**
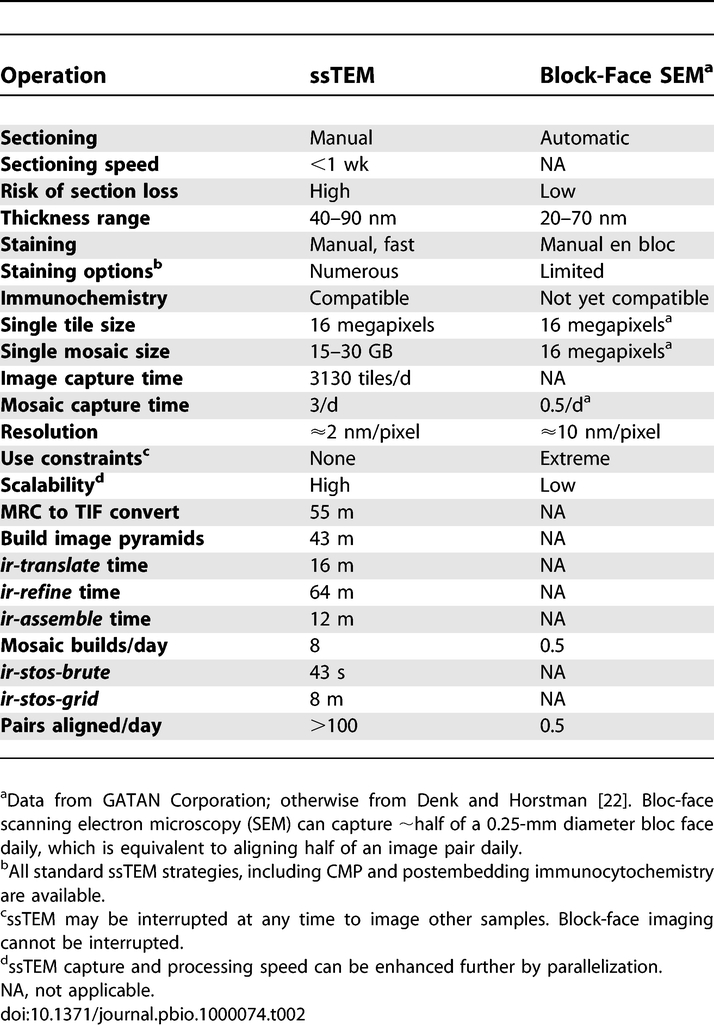
Platform Comparisons for a 250 μm Diameter x 30 μm High Volume

### The Challenge of Network Diversity

**Figure 1:**
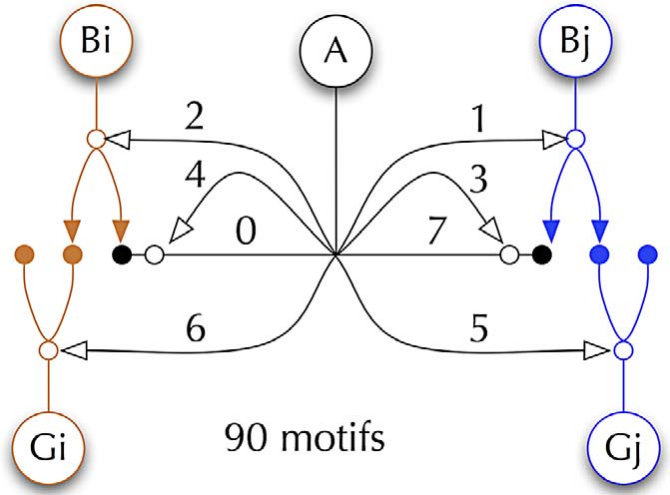
Neuronal Elements for Building Networks (A) Five-element micronetwork involving one AC (A) mediating cross talk between two vertical channels (i, j) with BCs (B) synaptically driving GCs (G). There are eight discrete AC connections (0–7), and the network can be configured in 90 formal motifs, at least 40 of which are of biological significance. Solid dots and arrows are excitatory; open dots and arrows are inhibitory.

Is ssTEM really necessary? Why can't we deduce networks from physiology, confocal imaging, or behavior? The answer is that potential network motifs derived by these methods are not unique. Diversity in potential network topologies is so high [17,26] that only anatomical ground truth can produce a valid connectome [17]. For example, mammalian retinas are simpler than those of most other vertebrates [27], but even so, no fewer than 70 unique cell classes exist [28]. And though the flow of signals from cone photoreceptors to ganglion cells (GCs) involves stereotyped networks that seem simple [29], a vast number of synaptic motifs can be produced from even a limited neuron set [17]. A small network of two different bipolar cells (BCs) driving two GC channels, interconnected by one amacrine cell (AC) class can be connected in 90 formal motifs and at least 40 of these are biologically tenable (Figures 1, S1, and S2). This is further compounded by unknown synaptic weights, molecular diversity of receptors and channels, gap junction display, and electrotonic constraints. With electrotonic constraints, the geometric locus of a synaptic contact matters [30–32] and the number of structural motifs possible in the simple five-element example extends to at least 640 (Figure S1). In stark contrast, we know that the outflow of signals from the mammalian retina is represented by only 15–20 GC classes, each representing a discrete filter channel [5,33,34].

### Anatomy Uncovers Unique Network Motifs

An anatomical approach that unambiguously determines motifs is required. This is justified by the fact that the efficacy of anatomy discovering complex motifs is unrivaled. Mammalian night (scotopic) vision is a prime example. The main scotopic signal flow network is rod → rod BC → rod AC, which then bifurcates into two synaptic arms that reenter the ON- and OFF-cone BC pathways. This motif was reported in 1974 by Helga Kolb and E.V. Famiglietti Jr. using ssTEM [12]. Subsequent physiological and genetic analyses [35,36] provided correlative support for the anatomical model, but neither study would have uniquely yielded the correct topology. Moreover, both transmission electron microscopy (TEM) [12,37–40] and light microscopy (LM) imaging studies [41,42] reveal that this network is even more complex. There are, in fact, no physiological data that either explain or predict these network submotifs. And despite five decades of robust physiology of retinal rod signaling, the discovery of a second scotopic pathway was also based on ssTEM [43,44]. Finally, corruption of scotopic motifs in retinal disease was discovered by TEM [45], again despite decades of electroretinographic analysis. It is unlikely that the day of ultrastructural discovery is past and we argue that it is just dawning.

### Requirements for Building CN Maps

Manually acquiring even small maps by ssTEM requires Herculean effort and high technical skill [21]. The gold-standard for such mapping has long been the Caenorhabditis elegans (C. elegans) ssTEM reconstruction project [46–50] where over 300 neurons, over 6,000 synapses, and nearly 900 gap junctions were traced through several instances of 1,000–2,000 section series, initially aligned and manually marked-up using the cinematographic method of Levinthal and Ware [51] developed in the early 1970s. While the actual build and analysis times are not available, we will show that a typical vertebrate brain canonical volume (see below) involves one to three orders of magnitude more connections and vastly more complex topology, since most of the C. elegans sections involve tracing linear tracts. The brute force manual method is simply impractical for building CN maps of more complex neural systems.

Three essential factors in building CN maps are (1) proper resolution, (2) statistical coverage, and (3) complete classification.

Resolution must be sufficient to unambiguously identify synaptic contacts and gap junctions [21] but not so high as to be unmanageable: nominally 2 nm/pixel. This yields synaptic vesicles spanned by 8–10 pixels that are robust for circuitry tracing.

**Figure 2:**
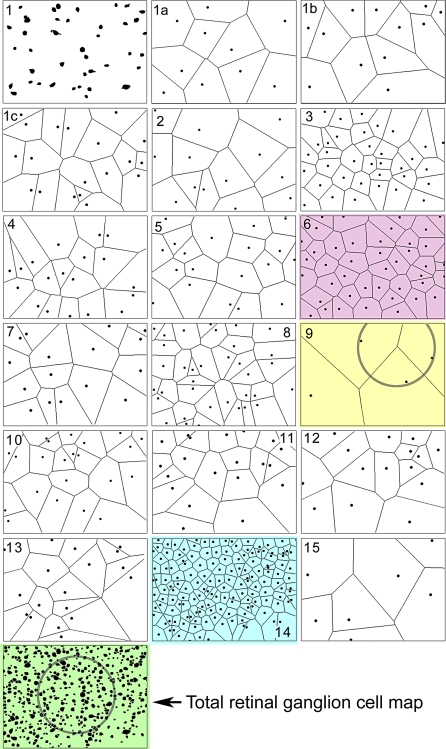
Voronoi Tiling of GC Classes in the Rabbit Retina Panels 1–15 represent GC or AC groups defined by CMP [5]. The bottom panel is the aggregate GC pattern. Each class forms its own independent tiling. Class 6 (red) is the most orderly. Class 9 (yellow) is the rarest and defines a canonical field for the GC cohort, containing three members of the class. Class 14 (cyan) is the densest and is the starburst displaced AC population. The canonical field superimposed on the entire GC cohort (green) captures >100 cells and guarantees a robust sample of all network types. Modified from Marc and Jones [5].

Coverage scales with neuronal diversity and density: a canonical region must be sampled. We define two coverage units (Figure 2). A *canonical tile* is bounded by the Voronoi domain (see Reese [11]) of the rarest neuronal element in a cellular array. In retina, this might be the dopaminergic polyaxonal cell [52] or OFF α GCs [5]. A slightly larger element is the *canonical field,* bounded by three somas (in planar systems such as retina) or four somas (in brain volumes) of the sparsest neuronal class. This ensures inclusion of multiple somas of all element classes in the region. However, not all canonical fields are known a priori and, arguably, molecular and anatomic data are key prerequisites to defining such domains. As we will show, a canonical field of retina can be acquired and assembled in less than 5 mo. Cortex is more challenging. Using normal primate ocular dominance column dimensions (>0.5 mm) and assuming a canonical repeat of 0.25 mm, the acquisition of V1 visual cortex would take 12 y. Reducing resolution to 10 nm/pixel (similar to bloc-face imaging) reduces acquisition to less than 1 y and new high-throughput TEM configurations (e.g., the Harvard Connectome Project) are allowing much larger capture fields, with as much as a 10× improvement in speed. Thus even canonical high-resolution cortical volumes are possible with ssTEM.

Classification (i.e., neuronal phenotyping) must be sufficiently robust to identify most major elements in a canonical field and may be the most effective way of identifying a field in the first instance [5]. It is not practical to decipher networks from large reconstructions when the number of classes of neurons is unknown. In general, panels of markers compatible with mapping networks are applied to or expressed in regions of interest [2,53]. In conjunction with serial section light microscopy (ssLM), we use computational molecular phenotyping (CMP, see Marc, Murry, and Basinger [54] and Marc and Jones [5]). CMP is a high-resolution optical imaging strategy that exploits sets of immunoglobulins (IgGs) targeting small molecules, precise multichannel image registration, and cluster analysis to extract defined neuronal classes in any tissue [5,27,54,55]. Small molecule optical CMP is compatible with ssTEM and enables classification of neuronal elements via direct multimodal image registration [17,56]. A tutorial on CMP is available in Protocol S1. In summary, building a CN map of any neural region such as retina requires canonical field imaging, neuronal phenotyping, image mosaicking, image registration, and image annotation. In this paper we detail complementary applications that can be used with a variety of datasets to produce large, high-resolution, aligned mosaics.

## Results

**Figure 3:**
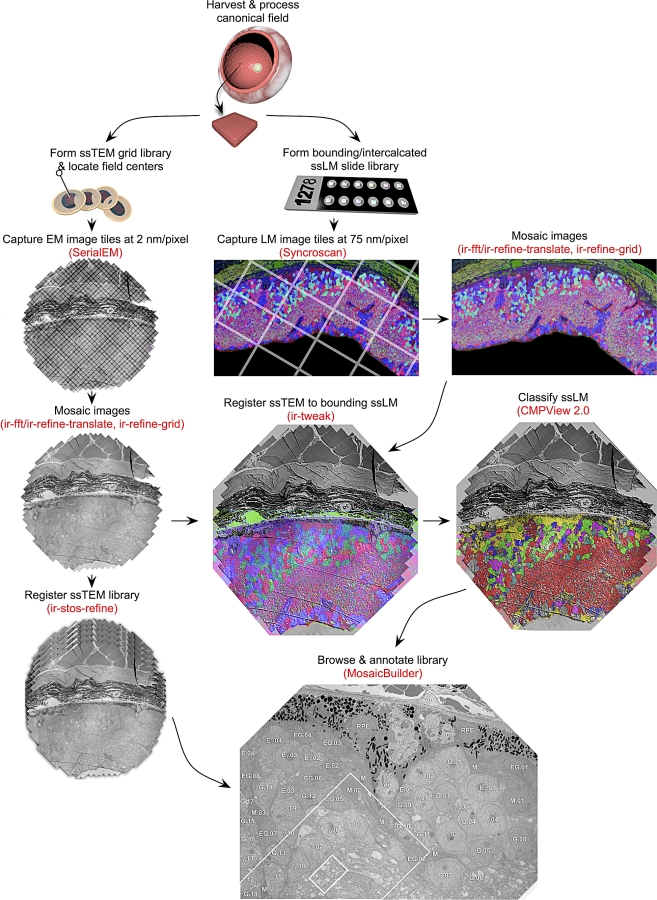
The Workflow for the ssTEM Ultrastructural Circuitry Framework Parallel serial section grid (ssTEM) and slide (ssLM) libraries are built. The ssLM libraries define either the bounds of the canonical field or are intercalated. Each library is acquired as a set of tiles mosaicked by *ir-tools*. ssLM and ssTEM mosaics are registered by *ir-tweak* and ssTEM volumes built with *ir-stos* applications. CMP classified ssLM imagery is merged with the volume to tag neurons and processes. The volumes are browsed with *MosiacBuilder/Viking* for process tracking and annotation.

We present here our complete framework and workflow, focusing on the image acquisition and processing pipeline for neural network mapping, with examples of image capture, mosaicking, and registration; fused molecular and ultrastructural data; volume construction; and browsing/annotating large datasets. The mapping workflow is built around a suite of image registration tools (*ir-tools*) and involves 12 basic steps (diagrammed in Figure 3): (1) harvest target tissue; (2) process for optimized CMP and electron microscopy; (3) section correlated ssTEM and ssLM libraries; (4) capture image tiles (*SerialEM*, Syncroscan); (5) build mosaics (*ir-fft*/*ir-refine-grid*, *ir-translate/ir-refine-grid*); (6) register ssTEM to bounding ssLM sets (*ir-tweak*); (7) classify ssLM sets (*CMPView*); (8) build ssTEM volumes (*ir-stos-brute*, *ir-stos-grid*); (9) register ssTEM processes to intercalated ssLM classes (*ir-tweak*); (10) visualize and track processes in the volume (*MosaicBuilder, Viking*); (11) tag synaptic connections (*Viking*); (12) summarize local circuitry data into network maps.

The collection of software tools for steps 1–12 are summarized in Table 1 and all are available for download. Detailed discussions of the algorithms referenced here are available in Protocol S1. This framework is obviously not restricted to analysis of the nervous system or any TEM or scanning electron microscopy (SEM) platform, but analyses of synaptic connectivity specifically require a characteristic resolution and canonical field, as will reconstruction of any other tissue volume.

### Acquiring Mosaic Tiles Manually

Many TEM facilities lack automated montaging, but this does not mean that high quality imagery cannot be obtained. Standard TEM imaging with sufficient image overlap can be obtained manually and images scanned at high resolution and bit depth. We use magnifications ranging from 5,000× to 10,000× and a typical manual ssTEM project size would be 100 image tiles per section. Similarly, corresponding bounding or intercalated ssLM sections can be imaged manually and require only a few tiles even at high resolution. However, as the positions of each ssLM and ssTEM image tile in the original sections are often lost, software tools to provide precise mosaic alignments are necessary.

### Acquiring Mosaic Tiles with *SerialEM*

Larger ssTEM datasets can be captured with automated imaging, exceeding 1,000 tiles. Such montaging requires robust control of stage position, camera behavior, metadata collection, and efficient use of resources. All of these are available through use of *SerialEM* software developed at the University of Colorado. *SerialEM* allows the use irregular capture patterns. No further user attention is required once all sections on a grid have been queued, which allows one to utilize the TEM during commonly idle night and weekend periods. In all regards we have found automated capture very resource efficient compared to manual approaches. Our current configuration captures 3,000 tiles in a day. The expanded capacity of ssTEM imaging requires a corresponding automation of ssLM tile collection. There are a number of commercial microscope tiling stages and our initial experience showed that the highest precision stages were essential to building mosaics of sufficient quality for CMP. However, the development of software tools for building mosaics informed by but not dependent upon stage metadata makes the X-Y precision of the stage less critical as long as overlap is adequate.

### Building Mosaics with *ir-fft*, *ir-translate*, and *ir-refine-grid*

**Figure 4:**
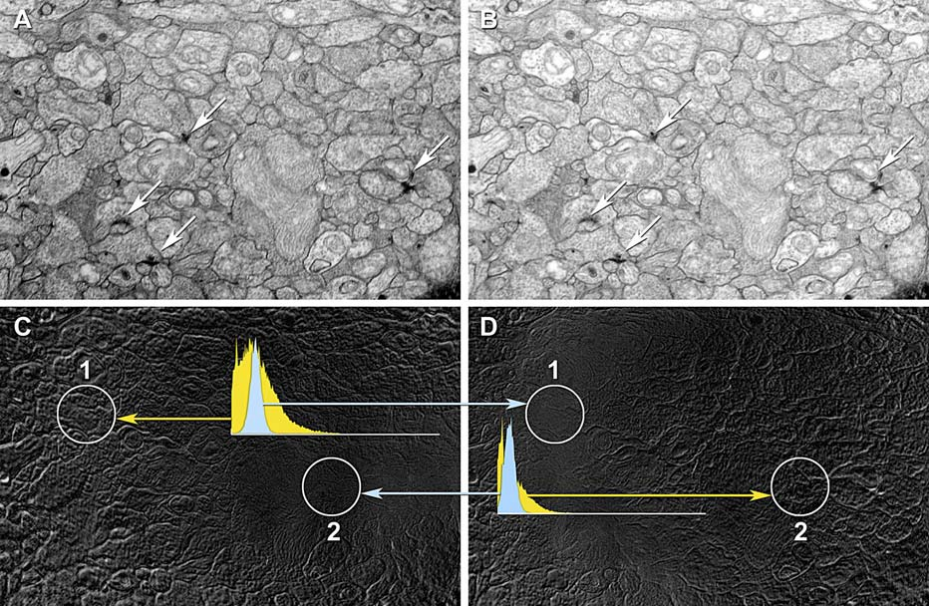
Distortions in Overlapping Tile Regions Visualized on Film Capture of ssTEM Data A typical low magnification (3,000×) field for synaptic screening in the rabbit inner plexiform layer was captured on a Hitachi H-600 with film images at ≈25% overlap. (A and B) represent part of the overlapping fields with slightly different densities due to the auto-exposure of different images. (C and D) represent the difference of (A and B) after translational/rotational best alignments for two regions (circles 1 and 2). When imagery in circle 2 is aligned best (C) the regions in circle 1 are shifted and have a higher image dispersion. The same is true when imagery in circle 1 is aligned best (D). The quality of alignment is quantified by normalized intensity histograms of corresponding patches. When spots are well-aligned (blue) the histograms are narrow, when poorly aligned (yellow) the intensity variance is high. The two spots are 6 μm apart. The histograms are peak normalized pixel number (ordinate) versus pixel value (abscissa, 0–255). Arrows indicate various ribbon and conventional synapses.

There are several challenges in ssTEM or ssLM image mosaicking with manual tile acquisition. First, every section exhibits an unpredictable rotation when placed in the TEM or on a slide and the number of tiles in each scan-line will differ. Thus it is typically not known which tiles are neighbors in a section. We developed the *ir-fft* algorithm to deduce the tile ordering automatically*.* The next challenge is the correction of nonlinear warps introduced into each tile from variations in electron imaging quality (Figure 4; also see Methods and Materials). Our solution finds pairs of overlapping tiles, computes their relative displacement, deduces a tile ordering, builds a layout of the mosaic without nonlinear warping, and refines the mosaic by applying nonlinear warps to each tile.

Ultimately, it is more efficient to build ssLM and ssTEM mosaics when coordinate information is available in the image metadata and the tool *ir-translate* exploits this. Only overlapping tiles are matched using the Fourier shift method *(ir-fft)*. This reduces the complexity of the method from a quadratic to a linear function of the number of tiles. Next, we define a tension vector proportional to the offset between the approximate position, and the preferred position as found by matching. These tensions are relaxed by iteratively moving the tiles.

**Figure 5:**
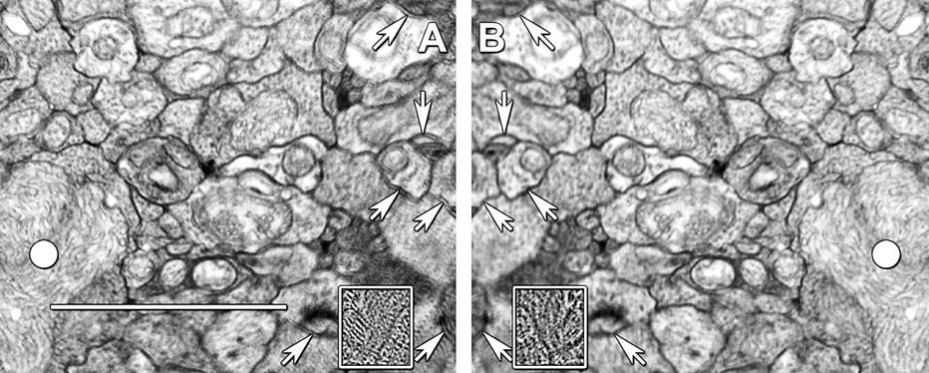
Recovery of Distortion Errors in Tile Overlaps with *ir-fft* and *ir-grid-refine* (A and B) are mirror images of the transparent overlays of fully overlapping regions of two tiles, both with best alignment centers on a large mitochondrion (white spot). (A) was auto-registered by *ir-translate* and many membranes appear as double images (arrows) due to nonlinear image distortions between the image pairs. (B) was registered with *ir-grid-refine* yielding improved membrane definition, even at very low magnification (resolution is about 5 nm/pixel, which accounts for the blurring). This is a worst-case scenario. With higher resolutions (more pixels) recovery is even more effective. The inset panels are high-pass 3 × 3-pixel filtered patches of the same region, showing severe moiré defects in (A). Scale = 2 μm.

Regardless of tile placement, most mosaics require some nonlinear warp refinement and this is accomplished with *ir-refine-grid*, an approach that places a course triangular mesh over each tile. Vertices are repositioned by registering their immediate neighborhood to overlapping tiles with the same matching algorithm utilized by *ir-fft*. Details of algorithm development are in Protocol S1. Each tile is sampled onto a coarse uniform triangle mesh, and small neighborhoods are sampled from all of the tile neighbors in the mosaic and the best matches determined as in *ir-fft.* A simple example of the ability of *ir-refine-grid* to manage subtle distortions is shown in Figure 5, where the TEM image tiles previously shown intractable under translation are readily aligned without user intervention. These are low resolution tiles: a worst-case scenario.

**Figure 6:**
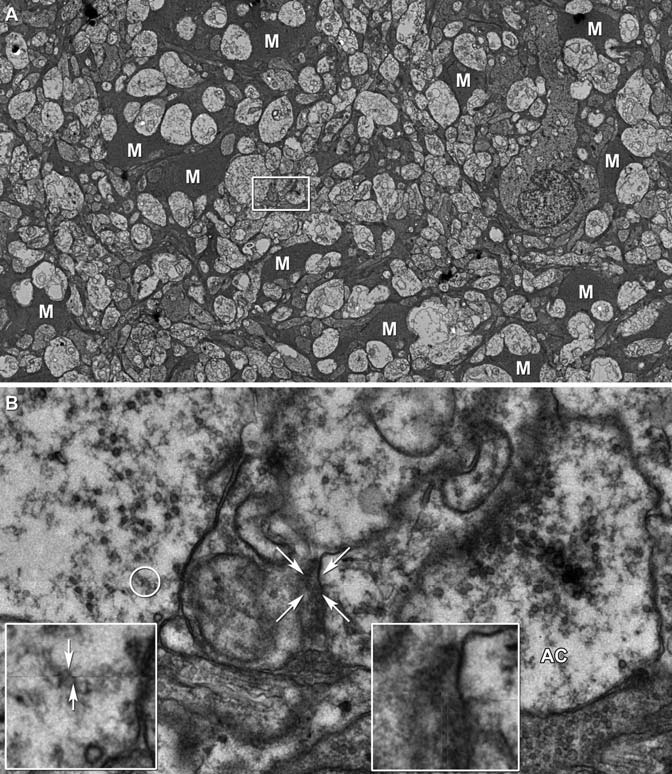
Representative Tile Overlaps Randomly Selected from a 1,000-Tile Array (A) A randomly selected region of rabbit retinal inner plexiform layer displaying parts of section number 105 containing 28 overlapping tiles. The overlaps are invisible at this magnification. Image width = 46.6 μm, M Müller cell processes. (B) Randomly selected boxed region from (A) containing tile overlaps, width = 4.76 μm. Arrows indicate a corner region among four tiles. A pair of vesicles (circled) is enlarged in the inset at left showing a misalignment between upper and lower tiles (arrows) corresponding to 7.8 nm or roughly one-third vesicle. The four corner region (arrows) is enlarged in the inset at right, showing no significant misalignment. The shaded margins of each tile are due to image processing edge enhancements. Most tiles have no measurable misalignment. AC, AC terminal.

Even when thousands of tiles are assembled, the alignment remains excellent. Figure 6 displays a randomly selected tile from a 275+ mosaic series of >1,000 tiles each, aligned with *ir-translate*. At the screen resolution used for synaptic markup, the tile edges are rarely visible. The error in alignment in this set of four overlapping tiles is extremely small, ranging from no detectable misalignment across three tiles to 7.8-nm shift in one tile: roughly one-third of a vesicle. Such errors are random rather than systematic through the volume, and do not accumulate.

**Figure 7:**
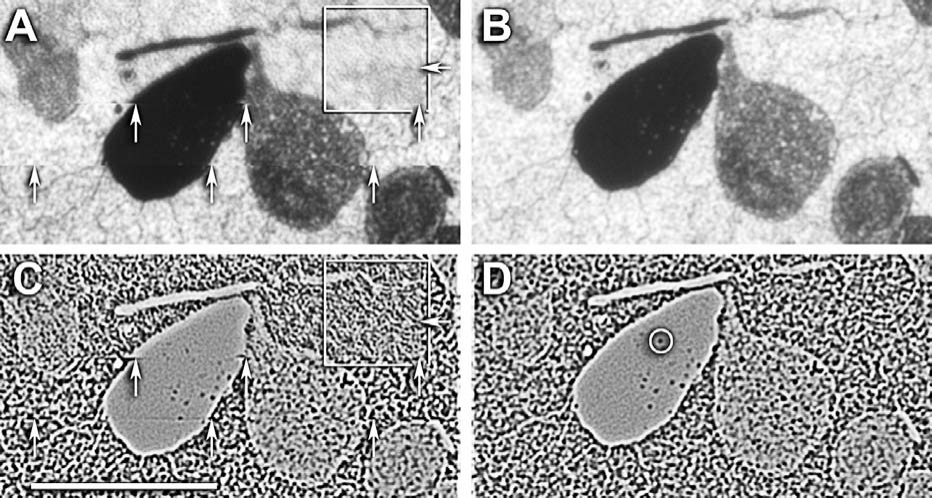
Auto-Registration of ssLM Image Tiles with *ir-translate* and *ir-grid-refine* A thin 200-nm section was probed with anti-AGB IgGs after excitation of the rabbit retinal GC layer [60], visualized by silver-intensified immunogold detection [54], captured on a SyncroscanRT montaging system (182 nm/pixel), and aligned with Syncroscan software (A and C) and *ir-translate/ir-grid-refine* (B and D)*.* At low magnification, both images appear perfect, but at near pixel level, many small defects emerge in the Syncroscan-aligned mosaic (arrows in [A and C]) that include 200–2,000-nm image shifts and image blurring (box). By using the raw image tiles and their metadata, *ir-translate* and *ir-grid-refine* create defect-free mosaics. While the image shifts shown in (A) are irrelevant (indeed invisible) for image display, they are highly corrupting in mathematically sensitive procedures such as clustering and multimodal alignments with ssTEM datasets. (A and B) are bright-field images and (C and D) are contrast-stretched Laplacian filtered images that enhance discontinuities and clearly show alignment defects. The circle in image (D) represents a lysosome of approximately 200 nm diameter. Its contrast is better preserved in the *ir-translate* and *ir-grid-refine* mosaic*.* Scale = 20 μm.

Together, *ir-tools* assemble superb mosaics. As an example, our Syncroscan system builds mosaics from arrays of LM tiles but invariably shows subtle misalignments or blurring at boundaries (Figure 7A). Conversely, *ir-tools* perform beautifully on exactly the same image tiles, generating seamless mosaics (Figure 7B). In truth, the Syncroscan errors are so small (≈200–2,000 nm) that they are generally invisible when the full image is viewed, but a Laplacian transform (Figure 7C) shows that there are many of them. When multiple channels are registered for CMP, these errors are additive, resulting in corruption of classification and ssLM-ssTEM registration. The LM images mosaicked by *ir-tools* are essentially perfect.

### Image Registration with *ir-tweak* and *ir-brute-stos*/*ir-refine-stos*

**Figure 8:**
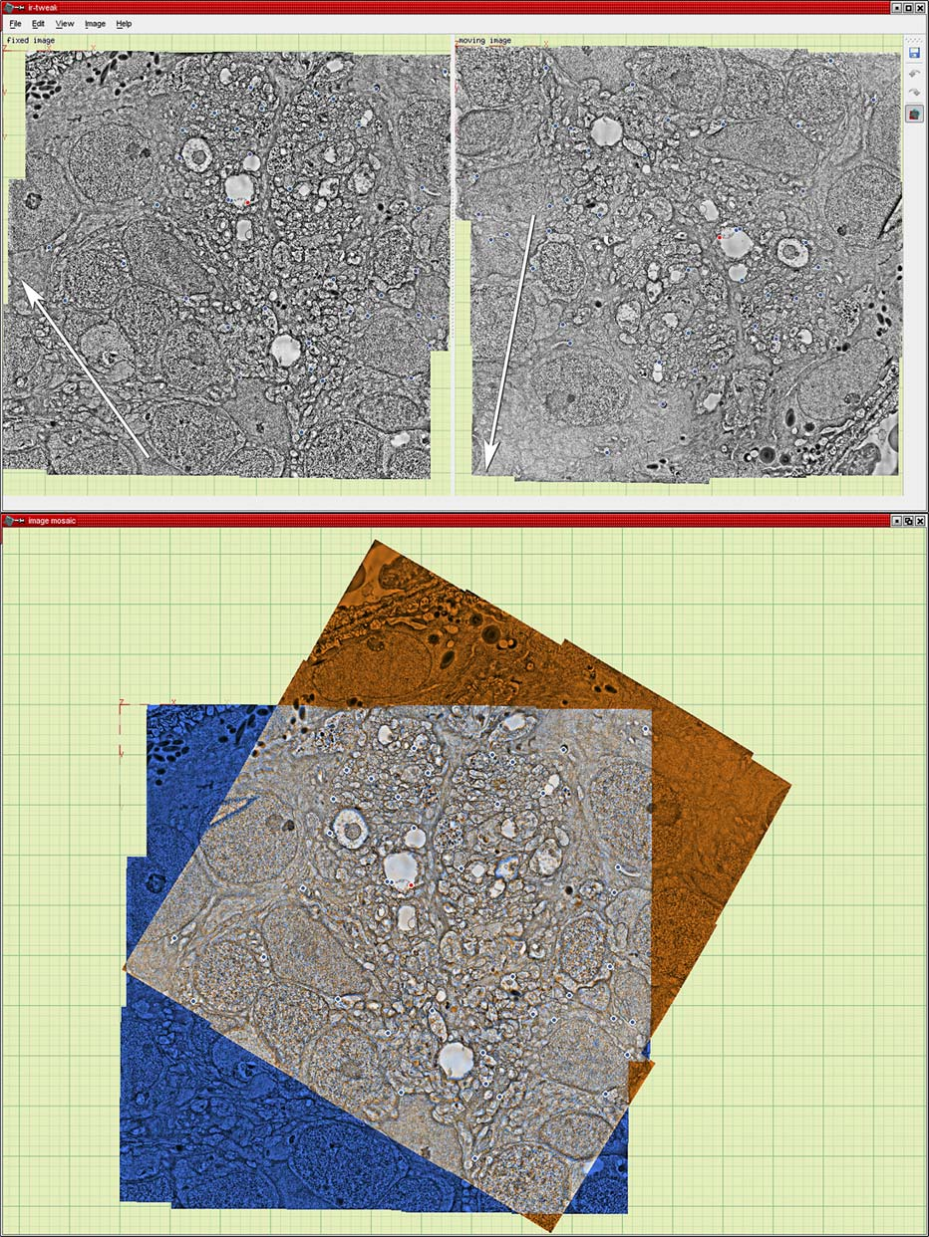
Registering ssTEM Image Tiles with *ir-tweak* The entire image represents two windows of the *ir-tweak* interface. The top window shows two serial sections from a manual film capture with tiles in different orientations (arrows), the left being the fixed and the right the moving or warped image. Successive control points (dots) entered on the fixed image by the user are predictively placed on the moving image based on the model calculated from all previous points, with a thin-plate spline strategy for accommodating local warps. The bottom panel shows the superimposed fixed (blue) and warped (orange) in real-time.

Both user-guided and automated image registration tools are needed for ssLM and ssTEM. User-guided applications are essential because some images (e.g., certain CMP imagery) lack sufficient information to drive automation. *Ir-tweak* is an interactive, multithreaded, cross-platform application for manual slice-to-slice registration. As control points are placed by the user in one image, their locations in the other image are estimated by the current thin-plate spline transform parameters. When the user corrects the locations of estimated points in the second image, the transform parameters are updated. Figure 8 shows the *ir-tweak* interface where the operator places points in the fixed image, adjusts their locations on the moving target image, and observes the registration dynamically.

**Figure 9:**
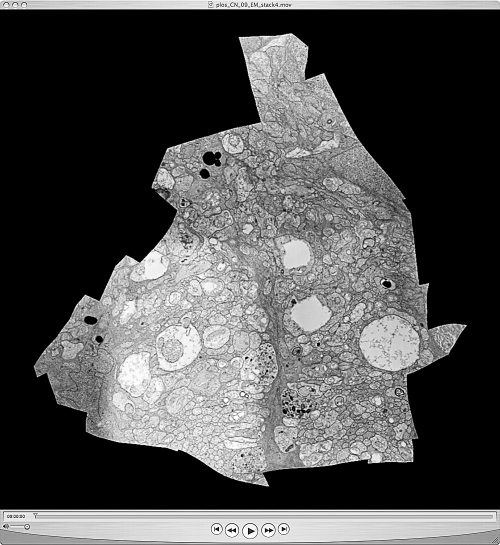
A Frame from a QuickTime Movie of a Volume Slice through a Mouse Retinal Microneuroma The microneuroma is 27 μm long and 16 μm wide at mid-length. The volume slice spans 45 sections, 90 nm each for a thickness of 4 μm. The original data were scanned from manually acquired TEM film images, aligned using *ir-stos* tools, and converted to a smaller movie using *ir-stom* (slice-to-movie), which generates 90 frames. Each slice is cropped such that only pixels with valid mapping onto every slice in the volume are kept. The raw serial image output from *ir-stom* was imported into QuickTime Pro v7 and saved as a movie. See Video S1.

While automated multimodal slice-to-slice registration remains an open challenge (see http://prometheus.med.utah.edu/∼marclab/gallery_CS.html for publicly available test sets), such boundary or intercalated registrations are manageable with user-guided tools such as *ir-tweak*. Conversely, automated ssTEM slice-to-slice *(stos)* image registration is essential to building volumes, even when image metadata are unavailable. As any section may be distorted by stretching or electron-optical defects, *stos* registration is similar to *ir-refine-grid*, with two differences. Since the orientation of slice pairs is arbitrary, we cannot use image correlation to estimate image-to-image translation parameters. Instead, we first perform a brute force search (*ir-stos-brute*) for tile translation/rotation parameters by downscaling the section mosaics to 128 × 128 pixel thumbnails and preprocessing (*ir-blob*) to enhance large blob-like features, preventing feature washout when downscaling. These parameters are then used to initialize the mesh transform at a fine resolution *(ir-stos-grid)* and applied to a “moving” slice relative to a chosen fixed slice. Figure 9 is from a down-sampled QuickTime movie (see Video S1) of a mouse retinal microneuroma ssTEM dataset acquired manually on film and automatically built into a volume of 45 auto-registered mosaics. This is a representative legacy dataset and is by far the most challenging type of data for automatic mosaicking and volume assembly due to lack of metadata and the presence of many section defects (stain artifacts, folds, dirt, beam burns). Even so, the alignment is excellent and suggests that many legacy ssTEM datasets can be exploited.

**Figure 10:**
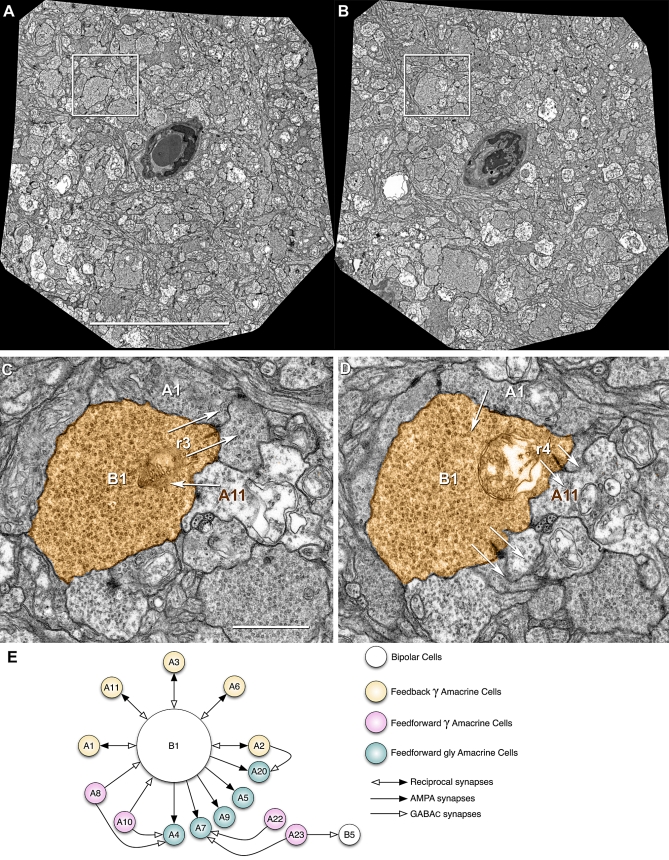
Automatic Neural Volume Assembly (A and B) automatically mosaicked slice 1 (A) and 10 (B) from an automatically registered 20-slice volume. Scale, 10 μm. (C and D) Slices 5 (C) and 9 (D) from the boxed regions in (A and B) showing that definition of reciprocal synapse identity requires ssTEM data. AC process A1 receives excitatory synaptic ribbon (r3) input from BC terminal B1 in (C), but does not show a feedback synapse until slice 9 (D). Similarly, AC process A11 show a feedback synapse in slice 5 (C) but does not receive excitatory synaptic ribbon (r4) input from BC terminal B1 until slice 9 (D). Arrows denotes synaptic polarity. Scale, 1 μm. (E) Partial summary of connections from markup of the volume. BC B1 drives five GABAergic (γ) ACs with reciprocal feedback and five glycinergic (gly) ACs. Four putative γ ACs provide feedforward inhibition onto some of the gly AC profiles. The origin of those processes is yet unknown.

**Figure 11:**
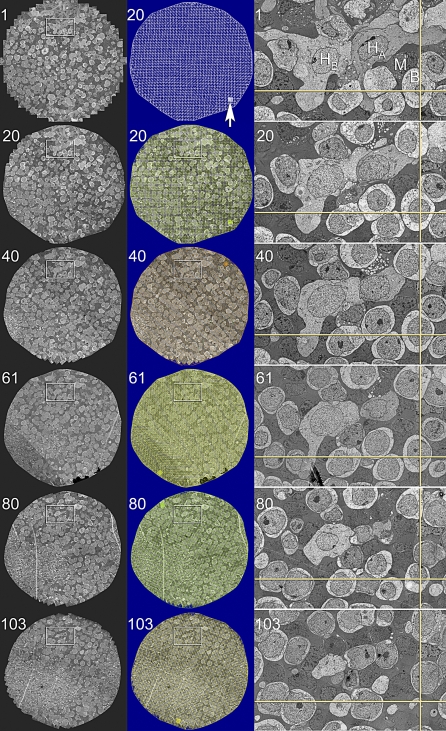
Automatic Registration of Canonical Scale Mosaics The left two columns are six 1,000+ tile mosaics from a series of over 120 horizontal plane 70-nm sections of the rabbit inner nuclear layer (sections 1, 20, 40, 61, 80, 103) spanning over 9 μm. Each mosaics is 250 μm wide. The middle column shows mosaics 20, 40, 61, 80, 103 with a colored overlay of the tile adjustment mesh (the true subtile mesh is much finer). The high contrast version of the mosaic 20 mesh shows that the bounding and bisecting lines only slight deviations from linearity due to slice-to-slice distortions. However, these do not accumulate. The arrow indicates a patch of the true mesh density. The right column (53 μm wide) is a magnified region of each slice showing the excellent cell-to-cell and subcellular alignment achieved by purely automatic image registration with *ir-tools*.

The availability of a large-format digital camera for TEM (e.g., the Gatan Ultrascan 4000) coupled with the most recent builds of *SerialEM* now make it possible to acquire large image fields at synaptic resolution from any specimen and begin assembling volumes automatically, such as the retinal circuitry volume for the rod BC layer in the mouse retina (Figure 10; Video S2). In this example, each slice was automatically mosaicked from 16 tiles (5,000×) with *ir-translate* and *ir-refine-grid* and a volume of 20 slices automatically registered with *ir-stos* (Figure 10A and 10B). Manually registering these datasets is impossible because of the many required distortion corrections among tiles and slices. With the *ir-tools* a year's manual work can be done in a day. Upon browsing the volume, characteristic connection motifs can be quickly extracted (Figure 10C and 10D) and graphically summarized (Figure 10E) from a text list of relations. Again, the goal is not to render 3D shapes, but rather browse and markup synaptic motifs. This volume readily detects characteristic reciprocal feedback, GABAergic local feedforward, and glycinergic long-range feedforward synaptic arrangements in the locale of the BC (see Video S3). Importantly, all regions are registered, not just the ones of local interest. We have similarly built volumes from 1,000-tile datasets of mouse retinal microneuromas (see below) and over 275 serial 1,000-tile mosaics from a 369-section series through the rabbit inner plexiform layer (Figure 11), with excellent automated alignment and without accumulating distortions. After automated registration through a volume of >100 sections (2 Tb), no error emerges from transforming all sections into the same volume space. While subtle slice-to-slice distortions exist due to physical deformation of sections, they do not accumulate and section-to-volume distortions are statistically indistinguishable from any those of any slice pair. Should such unlikely distortions emerge, our fast transform management method (see “Visualization and Annotation” below) allows the volume to be partitioned at any point and structures tracked across the parts. We can define break points and reference slices anywhere in the volume and rapidly create new series of transforms. This method is ideal for automated registration.

### CMP

**Figure 12:**
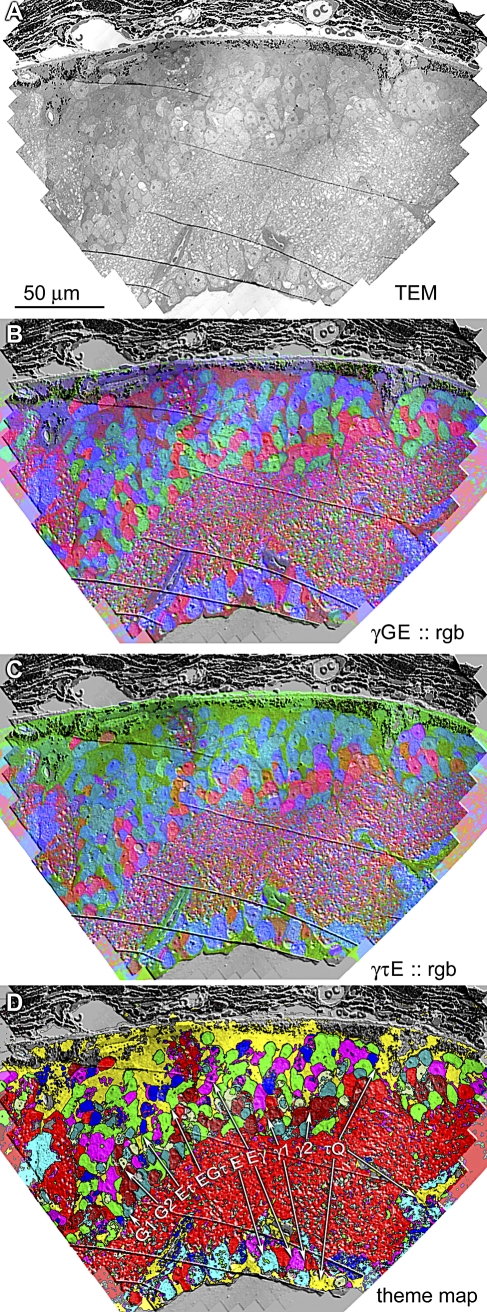
Fusion of ssLM CMP and ssTEM Data The mouse retinal microneuroma data shown in previous figures are comprised of the initial section in the ssTEM set (A), a set of four bounding 90-nm ssLM sections visualized by IgG γ, IgG G, IgG E, and IgG τ, all registered by *ir-tweak*, mapped as γGE :: rgb (B) and γτE :: rgb (C) triplets, converted by cluster analysis in CMPView into a classified theme map of nine discrete superclasses (see text).

CMP is a thin-section optical method that provides molecular signals for classification of cells and large processes. Ultrathin sections are immunoprobed for different small molecules, imaged optically, registered by *ir-tools*, and visualized as multichannel molecular signatures of different cell types. A tutorial on CMP is provided in Protocol S1. All cells have small molecule signatures and these are most evident in the central nervous system [57,58] and retina [5,27,54,59]. A library of four to eight small molecules can segment retinal populations into 20 or more natural molecular cell classes [5,55]. CMP can also segment many cell processes into different functional classes with high fidelity [17,56]. Figure 12 displays a retinal microneuroma ssTEM (Fig 12A), its bounding CMP ssLM images as multispectral overlays (Figure 12B and 12C), and its corresponding theme map after K-means classification with *CMPView* (Figure 12D). The four 90-nm sections preceding the ssTEM set were processed for CMP using IgGs targeting glutamate (IgG E), glycine (IgG G), taurine (IgG τ), and GABA (IgG γ) and aligned with the initial ssTEM image with *ir-tweak*. After classification with these four signatures alone, we show that there are four superclasses of ACs (γ1, γ2, G1, G2), two BC superclasses (Eτ, EτG), two GC superclasses (E, Eγ), the glial Müller cell class (τQ), and the retinal pigmented epithelium class, similar to results in normal mouse, primate, and rabbit retinas. One critical feature of such theme maps is completeness: every cell in the TEM mosaic is classified into a known biological group and every process traced from it is similarly tagged. No other method has yet achieved this scale of functional coverage.

**Figure 13:**
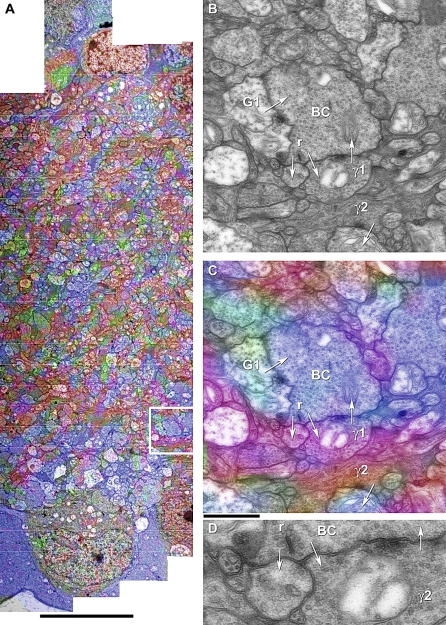
Fusion of ssLM CMP and ssTEM Data at the Synaptic Scale (A) A 20-μm wide strip from a 750-μm wide mouse retinal dataset of the inner plexiform layer extending from the AC layer (top) to the GC layer (bottom). The color map is a γGτ :: rgb mapping visualized as a transparency overlay onto the TEM data. Scale 10 μm. (B) The synaptic terminal of an ON cone BC (as identified by its signature and the region of the inner plexiform layer from which it was sampled, outlined in [A]). Four synapses are marked by arrows. The shaft of each arrow originates in the presynaptic process and the arrowhead lie in the target process. The BC is presynaptic to two profiles at a ribbon synapse (r) and postsynaptic to profiles γ1 and G1. (C) The ssLM CMP overlay, showing the characteristic blue τ+ signature of BCs, two different red GABAergic profiles (γ1 and γ2), and the green glycinergic profile (G1). (D) Enlargement of the classic BC ↔ AC GABAergic reciprocal feedback synapse. Scale 1 μm for both (B) and (C), and 400 nm for (D).

On a larger scale, a 0.75-mm wide sample of the mouse inner plexiform layer was mosaicked and augmented with CMP at sufficient resolution to identify many synapses directly (Figure 13). An example of the value of CMP signatures in defining circuits is shown in Figure 13B, where an ON cone BC [59,60] is presynaptic to a class γ1 AC process, which also makes a reciprocal synapse back onto the BC. This is an archetypal feedback motif (see Figure 10), one of the most common in retina [17]. In addition a G1 glycinergic AC process is presynaptic to the BC. This illustrates the powerful segmentation possible with ssLM CMP, even at the ultrastructural scale, enabled by *ir-tweak*. But why isn't simply sampling random examples sufficient? As shown by Marc and Liu [17], one of the most common motifs in retinal signaling is the nested feedback synapse, yet its full topology is rarely observed without ssTEM reconstruction.

### Visualization and Annotation

**Figure 14:**
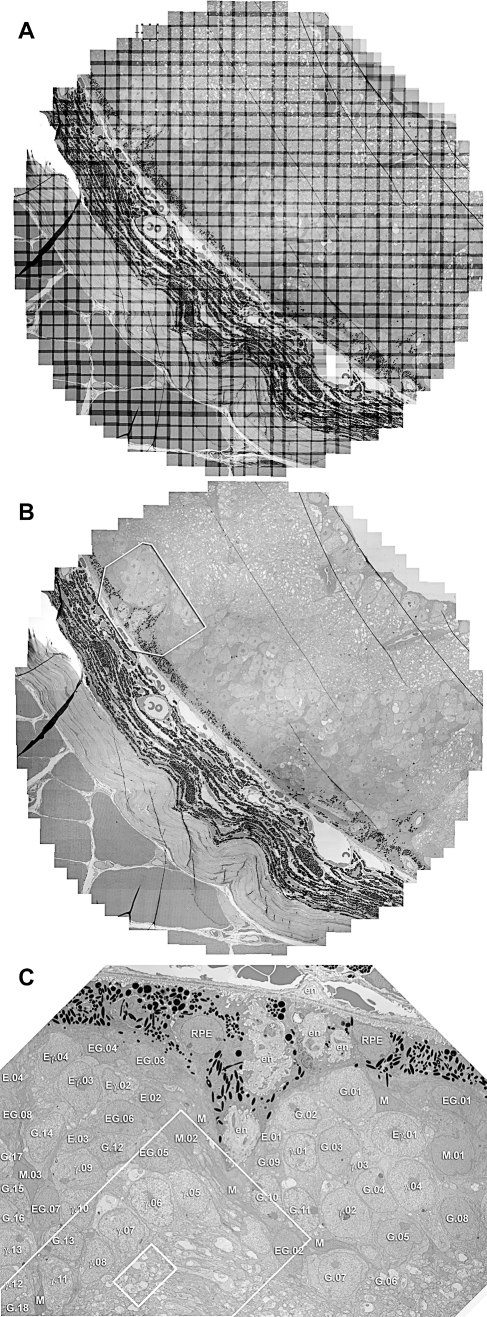
Browsing 30-GB Datasets with *MosaicBuilder* For this image, 1,001 TEM images were captured at 5,000× (2.18 nm/pixel) with *SerialEM*, tiled into a single mosaic dataset by *ir-translate* and *ir-grid-refine*, and visualized with *MosaicBuilder*. The overlap of each image with its neighbors is shown in (A) and the entire seamless image visualized in (B). The polygonal region in (B) is visualized in (C) simply by “zooming” in *MosaicBuilder* and the classifications obtained in Figure 11 annotated onto the initial section of the dataset. The rectangles in (C) are further enlargements that extend to the synaptic level. See Video S3.

Individual TEM mosaics can be many gigabytes in size, while final ssTEM volumes can be multiple terabytes. Exploring such large datasets requires new viewing tools. A single section can easily exceed the 32-bit limit (64 K × 64 K pixels) of most contemporary image file formats. Even if we exported full resolution mosaics to an image file for use with conventional imaging tools, each 8-bit grayscale 1,000-tile mosaic would require 16 GB of memory. This size is not yet common on desktop computers. To enable real-time viewing of the completed mosaics we used the established technique (e.g., Google Earth) of constructing an image pyramid for each tile and transforming them with the graphical processing unit. Only tiles visible on the screen are loaded and displayed at the needed resolution (Figure 14; Video S3). This technique makes the viewer memory footprint essentially volume-independent, providing several advantages. (1) Tile versions enhanced for contrast (*ir-clahe*), features (*ir-blob*), or any other processing can be substituted in real time by pointing the viewer at a different pyramid and using the same transforms. (2) Different transformations can be substituted to view results at each pipeline stage. (3) Reduced memory and bandwidth requirements of the pyramid-GPU approach make it possible for viewers to work over an HTTP connection. This is an important feature for collaborative annotation since the terabyte scale of the completed volume makes it difficult to relocate. (4) The transformations between volume and sections are known. Annotation loci can be moved from volume space back to section space for persistent storage, allowing one to update transforms or even reorder sections in the volume without losing the locations of established annotations.

*MosaicBuilder* is our completed Mac OS X viewer for viewing single sections and was our first visualization/annotation tool. *MosaicBuilder* imports the images files and transformation definitions generated by the *ir-tools* and then creates a single project file containing the image pyramid for the section and any annotations. A single logical file allows the final mosaic to be easily moved and shared among colleagues.

*Viking* is our web based volume viewer that allows the viewing of volumes over a reasonably fast internet connection. It uses the same image pyramid display strategy as Mosaic Builder, but instead of importing files into a single package, *Viking* reads an XML file containing HTTP links to all transforms and image files. *Viking* uses the slice-to-slice transforms (*ir-stos-grid*) to register all slices to a single reference section. The user can display any section in register with the volume and can easily page to adjacent sections to track structures. *Viking* also supports switching to view any grid transformation generated by the pipeline or alternate image pyramids generated by running image filters over the tiles.

### Scripting

As tools for the framework were developed, we were faced with the option to blend the tools into a single integrated application with a rich user interface or preserve each algorithm as a separate executable in a library of tools. We chose the latter as it is more flexible for code refinement and enhancement. However, by scripting each stage of the pipeline as a separate function in a Python package, we can invoke them with additional short scripts, automating pipeline execution. This process allows building the entire volume starting from raw microscope output using a single command. Data can be driven from any source into any stage of the volume building process via addition of a new function. The current scripting approach for building volumes does have a higher barrier to entry for new users compared to a single application. Though Python is not nearly as technical as the C++ environment used to create the *ir-tools*, changing the pipeline (e.g., adding support for a new microscope platform) does require some programming skill. The *ir-tools* have eliminated the most difficult technical challenges to volume construction, but the current state of the technology still mandates support from skilled programming personnel for the computational side of the reconstruction effort to be successful.

### Framework Parameters

Though sectioning and staining a 400+ section dataset is in itself a tour de force, it is well within the abilities of many ultrastructural laboratories and can be done in a few work days. And while even manual EM capture can take much longer than sectioning, multiplexing the task across several TEMs and operators also makes the task of acquiring ssTEM data practical. The image processing step has always been the real “show-stopper” when large scale ssTEM projects were conceived. Table 3 summarizes the canonical field, capture, and image processing parameters and timelines for a concrete project: a CN map of the rabbit retinal inner plexiform layer. This project specifies a resolution of 2.18 nm/pixel, which is sufficient to identify conventional/ribbon synapses and moderate scale gap junctions. In broad terms, an optimal canonical volume can be captured in about 3–5 mo with a complete volume build on a single machine.

**Table 3:**
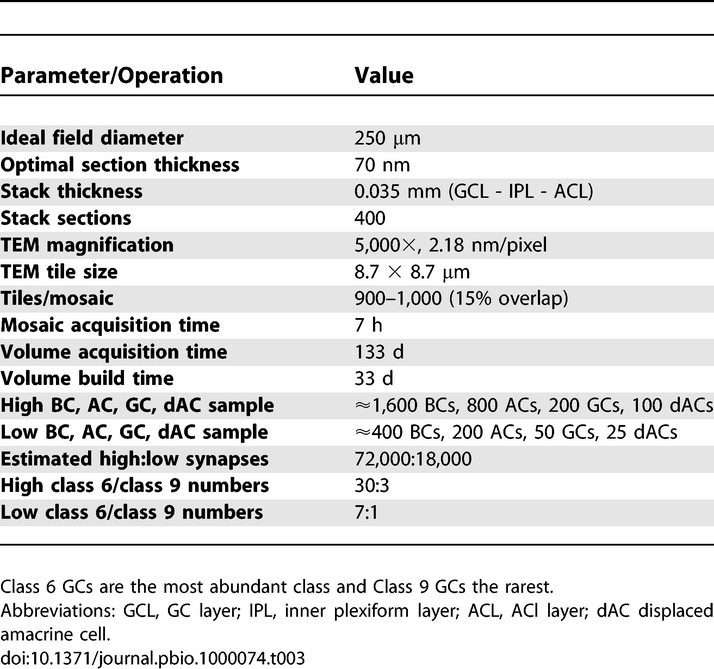
Parameters and Timeline for CN Mapping a Canonical Field in the Rabbit Retina

## Discussion

Our ultrastructural mapping framework removes three major barriers to large scale ssTEM reconstruction: mosaicking, registration, and viewing. While mathematically robust tools have long existed for analyst-guided nonlinear mosaicking and registration (e.g., PCI Geomatica; see Marc and Cameron [27]), and many solid efforts have been made to provide small-volume tools [19], the scale of ssTEM canonical volume reconstruction precludes a user-guided software solution. The ability of *ir-fft/ir-grid-refine* to automatically mosaic individual tiles and *ir-stos-brute*/*ir-stos-grid* to automatically register mosaics means that we have enabled any laboratory to build high-performance ssTEM volumes. Since scanned film imagery can be readily managed, we have also enabled volume construction and exploration of legacy datasets. Many extremely high quality ssTEM datasets have been produced in the past three decades [40,61–65], but their analyses have been restricted to one-time manual tabulations, drawings, and representative halftone imagery. Arguably, a key advance for anatomy would be the ability to allow global primary data access, similar to gene accessions. Our tools provide the framework for such global access via a central repository. And despite the development of early far-sighted reconstruction frameworks [66] and subsequent enhancements, the code, platforms, and throughput of those schemata reached neither the performance nor availability required for canonical field reconstructions.

### CMP and ssTEM

The importance of molecular classification of neural data cannot be overstated. Without even partial classification, ssTEM reconstructions remain of limited value. This observation remains true even with the ability to nominally identify individual cells by stochastic, multivariate protein expression [2]. In contrast, small molecule CMP allows the categorization of class partners in networks before the network is built from ssTEM. Classification by post hoc unraveling of connectivity is undoubtedly the most unwieldy and statistically challenging way to identify synaptic partners.

### The Retinal CN Mapping Framework

**Figure 15:**
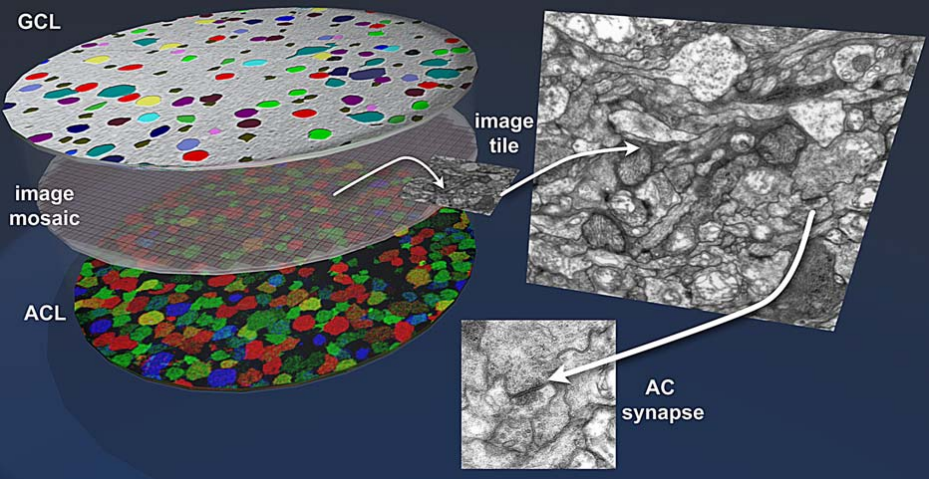
The Retinal CN Mapping Framework Canonical fields of rabbit retina are being sectioned from the GC to the AC layers at 70 nm and tiled mosaics acquired for volume assembly. Bounding the ssTEM set are classified sets of GCs (top) and ACs (bottom) whose processes enter the field and can be tagged and tracked. The GC patch is shown as a theme map and the AC patch as a γ.AGB.E :: rgb mapped image. At 5,000× it is possible to unambiguously identify both conventional and ribbon synapses as well as most gap junctions that exceed ≈200 nm in lateral extent. As CMP can be performed on sections as thin as 40 nm, selected molecular signals can be intercalated into ssTEM sets without significant disruption of volume builds by saving spaced sections for CMP, using them if needed, or reinserting them as ssTEM elements if not.

Our specific objective in developing these tools is retinal CN mapping. We have begun implementation of this process by developing a rabbit retinal preparation with strong image segmentation. As shown previously [5,60,67], augmenting CMP libraries with the activity marker 1-amino-4-guanidobutane (AGB) generates a nearly complete neuronal classification. These signals are also fully compatible with ssTEM [56]. We have prepared a single retinal preparation with 16 patches each defined as a canonical field for CN mapping. These patches are being sectioned, stained, and captured with an estimated completion date of mid-March 2009. The strategy uses horizontal serial sections (sections in the plane of the retina) beginning from either the AC or GC side of the inner plexiform layer (Figure 15). Those cellular layers are first classified as CMP bounding layers registered to the ssTEM set of >400 sections, with each section captured in mosaics of 950–1,100 tiles. Upon completion of each volume, it will be available for our own and community browsing and annotation, described as follows.

### A Proposal for Multi-TEM Projects

Most of the example ssTEM volumes our group has produced so far have been collected with a single high-performance microscope. We can capture 3,000 tiles/day. However, the install base of manual TEM systems or film-based systems with montaging stages far exceeds those with high-resolution digital cameras. Further, the performance of film is still superior to any digital system and the potential for capturing high bit-depth scanned images manually augmented with positional metadata makes our ultrastructural framework even more practical. By fragmenting large projects into packets of grids that can be captured in parallel, it is possible to speed tile acquisition multiplicatively and then distribute tiles to a central resource for volume builds.

The next phase of CN mapping is analysis: building a description of connectivity by tagging cells and processes and marking synapses. Our goal is not to render 3D ultrastructural images, but rather tabulate connections within the volume. While it is plausible to develop automated synapse and gap junction recognition tools (perhaps augmented by molecular probes), those tools are in early development stages. Our experience is that analysts can perform excellent tagging and synapse markup with these tools. Furthermore, large datasets can be analyzed in parallel by large groups. A wonderful example of this is the www.galaxyzoo.org project to classify millions of galaxies imaged by various platforms such as the Sloan Digital Sky Survey (www.sdss.org). Given the importance of mammalian central nervous system circuitry analysis in neurological disorders, the notion of a single lab performing cradle-to-grave processing on a system is increasingly impractical, as is the notion that computational pattern recognition can adequately screen data without missing important observations. Human eyes remain the best pattern recognition systems for ssTEM data. The value of our strategy to develop a scalable, web-compliant viewer for community markup lies in the fact that new, powerful acquisition platforms [23–25] and their descendants will soon create an additional deluge of high-quality data.

### Future Developments

Our next phases of development target six areas. (1) Auto-tracking: Computational techniques for segmenting and tracing individual neurons across a large number of ssTEM sections [68,69] are critical to speed network data collection. (2) Auto-markup: We are exploring a large library of identified synapses to train automate synapse markup and implement logical rules for synapse identification and signal polarity. These efforts will not replace human tracking and markup in the short term, but needn't be extremely efficient to accelerate analysis several-fold. (3) Enhanced pipeline speed: Even though we can speed mosaic and volume builds by using more machines, we still seek to vastly improve tool speed to accommodate larger canonical volumes. For example, a canonical volume in primary visual cortex spanning an ocular dominance column is several times larger than the retinal canonical volume. (4) Simplified pipeline integration: Our efforts to develop acquisition, classification, mosaicking, registration, browsing, and markup tools have originated with several developers using different platforms. How much multiplatform development is justified? Certainly we argue for an open, platform neutral code base for future development. However, data transport across platforms is now so simple that it is not essential to spend development resources in replicating applications. Rather, improved pipeline scripts and interfaces will be our short-term focus. (5) Volume viewing with http compliance: We are creating a volume slice-by-slice viewer that enhances the speed of synapse tagging for building CN maps. While not essential to our framework, it offers the ability to lever current web protocols to facilitate community markup. (6) Enhanced CMP power: We are screening libraries of macromolecules (e.g., Marc et al. [70] and Micheva and Smith [7]) for ssTEM-compliance to augment our CMP library.

Finally, the informatics challenges deserve mention. We are hardly alone in this venture. Many groups have addressed the informatics of neuroscience data collections, especially the need to aggregate resources, e.g., the Neuroscience Information Framework (http://nif.nih.gov), soon to be transferred to the supervision of the National Center for Microscopy and Imaging Research at the University of California at San Diego (http://www-ncmir.ucsd.edu/), and the International Neuroinformatics Coordinating Facility (http://incf.org). However, a key issue is a lack of multiresolution annotation tools and task administration for large-scale distributed, multiresolution datasets. Though large scale navigational tools have been built by the Allen Institute for Brain Science (http://www.brain-map.org/) and Brain Maps developed by Ed Jones and colleagues at the University of California Davis (http://brainmaps.org/), robust tools for community markup that can navigate high resolution TEM datasets have yet to be created or validated. Ontologies for neural systems are under rapid development (http://ccdb.ucsd.edu/CCDBWebSite/sao.html), which will be essential to building these markup tools. These and other groundbreaking efforts validate the need for next-generation software including rapid navigation of fused TEM-multivariate molecular data, true volumetric atlases, graph-theory based analyses, and dynamic ontology updating.

### Summary

High-performance ssTEM is a powerful technology coupled to traditionally artisanal data presentation and analysis methods. These are poorly adapted to large-scale collaborations or high-throughput screening. Many laboratories have attempted to develop stronger tools for ssTEM throughput, but most efforts were hampered by many barriers: code that did not scale, limited processor speed, expensive storage, and small canonical volumes. Our framework largely overcomes all computational barriers, providing highly standardized collaborative environments that enable ssTEM to serve as both a statistically practical CN mapping tool and an effective screening/phenotyping tool for modern neurogenetics.

## Materials and Methods

### Tissue harvest, processing, and sectioning.

All animal use including methods for anesthesia and euthanasia conformed to institutional animal care and use authorizations at the University of Utah and to the Association for Research in Vision and Ophthalmology Statement for the Use of Animals in Ophthalmic and Visual Research. Retinal samples were taken from Dutch Belted rabbits (Oregon Rabbitry), C57Bl/6J mice (The Jackson Laboratories), and TG9N transgenic mice that have an aggressive photoreceptor degeneration and neural remodeling defect [56]. Light-adapted adult male and female pigmented rabbits tranquilized with intramuscular ketamine/xylazine were deeply anesthetized with intraperitoneal urethane in saline, euthanized by thoracotomy in accord with University of Utah Institutional Animal Care and Use Committee guidelines, and the eyes immediately injected with 0.1 ml fixative and an additional 18-Ga needle pressure relief. Rabbit eyes were enucleated, hemisected, and fixed in 1% formaldehyde, 2.5% glutaraldehyde, 3% sucrose, 1 mM MgSO4, in 0.1 M phosphate or cacodylate buffer, (pH 7.4). Light-adapted mice were rapidly euthanized with halothane or an isoflurane vaporizer. Mouse eyes were slit at the limbus and injected slowly with 0.1 ml fixative before enucleation and immersion fixation for 24 h. All tissues were osmicated 45–60 min in 0.5%–1% OsO4 in 0.1M cacodylate buffer, processed in maleate buffer for en bloc staining with uranyl acetate, and processed for resin embedding as described in Marc and Liu [17]. Specifically for CN maps, flat resin mounts of retina are remounted for serial sections in the horizontal plane through the inner plexiform layer [27,71]. Vertical sections of mouse retina were used to define normal C57Bl6/j and disordered TG9N mouse retinal circuitries. Serial sections were cut at 60–90 nm with various models of Leica ultramicrotomes onto carbon-coated Formvar films on gold slot grids and imaged at 80 KeV in either a Hitachi H-600 or JEOL JEM 1400 electron microscope at 5,000–10,000× magnification. Images were captured directly on film (Kodak 4489 Electron Microscope Film) and digitized at 16 bits grayscale on Ultramax or Creoscitex scanners, or captured digitally on a GATAN Ultrascan 4000 16 megapixel 16-bit camera.

### Image capture.

Creating the CN map of the retina requires digitizing each tissue section and registering it to its neighbors. Creating a volume of this scale is a significant undertaking: The CN map for the rabbit inner plexiform layer in the visual streak requires a volume delimited by a canonical field 250 μm in diameter × 30 μm high: roughly 1.47 × 10^6^ μm^3^. A cylindrical volume is a more efficient capture object than rhomboidal prisms that will have extremities clipped out as sections are rotated during registration. While the issue is irrelevant at small volumes [18], it tremendously impacts beam time at canonical scales. In practice, at a magnification of 5,000× on the JEOL JEM-1400, we capture 950–1,100 images or tiles/section and ≈333 sections at 70–90-nm thickness. Storage of unprocessed 16-bit images requires 10.4 terabytes. With a time of capture at roughly 30 s/frame, this requires some 70–100 calendar days on a single TEM, which argues for automated capture scripts and efficient capture geometries. To ensure the images can be positioned properly in the total mosaic, each image has 15% area overlap with its neighbors. With some of the software tools developed below, it is also evident that such tasks can be parallelized across microscopes and users.

We capture ssTEM data using *SerialEM* software developed by D. Mastronarde at the University of Colorado at Boulder [72]. Though originally developed for TEM tomography, *SerialEM* is ideal for large-scale mosaicking. The most recent build version of *SerialEM* allows definition of multiple circular or polygonal regions of interest on a grid and automates stage drive and image capture within the regions of interest on the JEOL JEM 1400 TEM (and other recent TEMs as well such as FEI Tecnais) and, critically, stores stage position metadata for each tile. This greatly reduces the computational cost of the initial positioning of mosaic tiles from O(n^2^) to O(n). The program includes a scripting capability that provides the flexibility needed to optimize the acquisition strategy, for example, by focusing only on an appropriately spaced subset of the image tiles. While automated capture is ideal for the microscope's Gatan Ultrascan 4000 (4K × 4K) camera, it can also serve on a smaller scale with film capture.

### Mosaicking.

We use the same software suite (*ir-tools*) to mosaic ssLM and ssTEM data. In an ideal setting, we have stage position metadata for both kinds of datasets (from Syncroscan and *SerialEM*), which can be used by *ir-translate* to produce precise initial image mosaics. This is then followed by *ir-refine grid* to correct for image aberrations in each tile. However, some users may lack capture automation. In this more general case, an operator manually adjusts the position of each tile, aiming for a specified but often imprecise overlap between tiles. Even approximate coordinate information is often not recorded. Given a large number of tiles specified in no particular order, a mosaic must be constructed and individual tiles corrected for distortions, especially subtle electron-optical aberrations (spherical aberrations, magnification astigmatism, local heating motions, charging motions, etc.) that are often undetectable until precise alignment is attempted (Figure 4). It is rare that the magnification in the average TEM is exactly the same at the field center and edge. At a modest magnification yielding a 7.5-μm wide field (about 5,000×), a 1% error in magnification at the edge of the field (4,950×) yields a displacement of over three vesicles: potentially a massive misalignment in tracing circuits. A mosaicking scheme that addresses this general problem is *ir-fft* followed by *ir-refine-grid*.

### Image registration and volume construction.

Slice-to-slice image registration is further complicated by differences in imaging modalities (ssLM versus ssTEM), changing shapes of cells and processes, and physical distortions affecting individual sections (folds, stretching). At the boundaries of or intercalated within the ssTEM volume, we collect ssLM data for CMP analysis. Similar to the multimodal alignment strategies used by Marc and colleagues [5,56], ssLM images are operator-registered to adjacent ssTEM slices using *ir-tweak*. However, manually registering large ssTEM volumes is impractical and automated slice-to-slice tools are needed: (*ir-stos-brute, ir-stos-grid*).

### CMP and classification.

CMP is a method to extract quantitative molecular signatures from cells or even cellular processes [5,54] and fuse molecular signature data with ssTEM datasets [17,56]. ssLM samples (40–90 nm) are arrayed on 12-spot Teflon-coated slides (Cel-Line; Erie Scientific Inc), fully sodium ethoxide etched, probed with IgGs targeting various molecules, and visualized with silver-intensified 1.4-nm gold granules conjugated to goat anti-rabbit or goat anti-mouse IgG (Nanoprobes). Immunoreactivity in these samples is a pure surface phenomenon independent of section thickness [54]. The IgG library includes (but is not limited to) anti-L-aspartate (IgG D), anti-L-glutamate (IgG E), anti-glycine (IgG G), anti-L-glutamine (IgG Q), anti-taurine (IgG τ), and anti-GABA (IgG γ) (Signature Immunologics Inc.). All data were captured as 8-bit 1,388 pixel × 1,036-line frames under voltage-regulated tungsten halogen flux with a variation of 1.2 ± 0.6%/min (mean ± SD). Image mosaic tiles were captured with a Peltier-cooled QImaging Fast 1394 QICAM (QImaging) and a Syncroscan montaging system (Synoptics Inc.) on a Scan 100 × 100 stage (Märzhäuser Wetzlar GMBH & Co.). ssLM mosaics were prepared using various *ir-tools* and aligned using *ir-tweak* (see below). CMP classification (clustering, histogram analysis, PCA, etc.) is performed on the CMP dataset using *CMPView* (J. Anderson, 2008) to phenotype processes and cells. *CMPview* is built in MATLAB2008a. Clustering is based on the robust K-means and isodata algorithms (see Marc et al. [5,54]), augmented with interactive histogram splitting tools. *CMPView* operates on either pixel-based or object-based images. The intermediate product of relevance for this paper is the production of a high-resolution theme map of cell classes that is then registered to ssTEM data [17,56]. Finally, image analysis for characterization of mosaicking and registration performance was performed using ImageJ [73].

### Image browsing and annotation.

Once large mosaics are built, it is essential to have tools to browse, annotate, and record annotations. Some mosaics are 15–30 GB datasets, which are unmanageable by most commercial imaging tools. We have developed two applications, *MosaicBuilder* (Mac OS X) and *Viking* (Win), that allow single slices to be viewed, browsed, and annotated. By using image pyramids, these tools quickly navigate between low and high magnification views. Volumes are even more challenging, as they expand into terabyte size and *Viking* allows rapid paging through the build over an HTTP connection, enabling single image repositories to serve multiple users.

### Managing the processing pipeline.

This 12-stage framework requires significant user management and exceptional digital hygiene in data archiving, access, and revision control. Since each computational stage invokes a different program and occasional transitions between data formats we elected to use the Python scripting language and Python Imaging Library to bridge stages. The scripts perform tasks such as conversion from microscope-specific formats to the plain text “.mosaic” format of *ir-tools*, image cropping, contrast enhancement, down-sampling, and launching multiple instances of single-threaded algorithms to ensure each CPU core is fully utilized. Pipeline automation improves throughput, eases integration, produces consistent results, and stabilizes performance.

## Supporting Information

Figure S1 How Many Network Motifs Can You Make with Five Retinal Neurons? Assume that you have five kinds of cells: two different BCs (Bi, Bj), two different GCs (Gi, Gj), one AC (A) connecting them.
The required vertical channels are Bi → Gi; Bj → Gj. The lateral channel options are: A → none: processes 0 and 1; A → Bi, Bj (feedback): processes 2 and 3; A → Gi, Gj (feedforward): processes 4 and 5; A → A (nested feedback): loop x.
We start with a summary of submotifs. There are 12 allowed BCAC submotifs: motif → label; 0 → i mono input, no feedback; 1 → j mono input, no feedback; 01 → dual input, no feedback; 02 → i mono input, in-channel feedback; 03 → i mono input, cross-channel feedback; 12 → j mono input, cross-channel feedback; 13 → j mono input, in-channel feedback; 012 → dual input, feedback I; 013 → dual input, feedback j; 023 → i mono input, dual feedback; 123 → j mono input, dual feedback; 0123 → dual input, dual feedback.
There are four possible AC → GC feedforward submotifs. A → none; A → Gi; A → Gj; A → Gi and Gj.
There are two AC → AC nested feedback submotifs. A → none; A → A.
The total combined motif number is ((12 BC → AC)(4 AC → GC) − 3)(2 AC → AC) = (45 basic submotifs)(2 nested forms) = 90.
In connective terms this calculation includes −3 because motifs 0, 1, 01 cannot also have AC → no GCs. However, there are AC volume release mechanisms (peptides, NO, monoamines) that could have this motif. In a strict biological sense, there could be least 96 motifs. But in practice we might require constraints (see Figure S2). How many network motifs can you make with five or six retinal neurons?
(1.29 MB TIF)

Figure S2 How Many Motifs Can You Make with Five or Six Retinal Neurons? Even if we add the strong biological constraint that a minimal submotif must have a path to both GCs and one cross channel AC path (i.e., Bi → Ai → Bj), there are still many possible motifs. Here we redraw the pattern in Figure 1 to simplify the analysis.
(A) This is the complete five-neuron network. Three connections (5AA, 4Ai, 4Aj) can be independently removed: 2^3^ = 8 motifs are possible with these connections varied.
(B) After removing 5AA, 4Ai, and 4Aj we have a basic submotif (1) that can be decimated by removing connection pairs. Submotifs 1–5 satisfy our constraint, thus admitting 40 biologically likely networks. Submotifs 6 and 7 are biologically plausible, but don't form a single network.
What happens if we add one more AC?
(C) This is the complete 40-motif network.
(D) If one more AC is added (Aj), it represents an independent 40-motif network and the total possible paths becomes 40^2^ = 1,600.
(E) By adding connections between Ai and Aj increases the number 4-fold (none, Ai → Aj, Aj → Ai, Ai ↔ Aj) or 6,400. Thus the diversity of possible connections is so high that the most effective way to discover neural circuits is to actually map them by ssTEM.
(KB TIF)

Protocol S1 Details of Image Processing Tools and a Tutorial on CMP (836 KB PDF)

Video S1 A QuickTime Movie of a Volume Slice through a Mouse Retinal Microneuroma See Figure 9 for details.
(4.30 MB MOV)

Video S2 QuickTime Movie of an Auto-Registered Synaptic Volume See Figure 10 for details. This movie is a 20-slice series (looped back and forth) through BC terminal B1 (highlighted in orange) shown in Figure 10 of the manuscript. Each instance of a BC synaptic ribbon in B1 is denoted by an aqua dot for the duration of the ribbon across slices.
(3.28 MB MOV)

Video S3 QuickTime Movie of the *MosaicBuilder I*nterface See Figure 14. Here we zoom to the synaptic level with structural markups (captured in real-time by iShowU, http://shinywhitebox.com). Tiles from a vertical section of rabbit retina prepared for assessment of CN capture parameters were captured at 5,000× with *SerialEM*, pipelined through *ir-translate* and *ir-refine-grid* into a large mosaic and visualized in *MosaicBuilder* for synaptic markup. The movie shows slider-based zooming of the dataset from a moderate scale up to synaptic level, first to a characteristic AC → GC synapse in the OFF sublamina. The synapse is marked by a red pin in the synaptic cleft and an arrow denoting polarity. These markup metadata are recorded in an *.xml export. The field is then zoomed out and repositioned to show a characteristic AC → ON cone BC synapse. Two blurring events in zooming represent the transitions in resolution in the image pyramid scheme.
(9.55 MB MOV)

## Abbreviations

AC: amacrine cell
AGB: 1-amino-4-guanidobutane
BC: bipolar cell
CMP: computational molecular phenotyping
CN: complete network
GC: ganglion cell
IgG: immunoglobulin
LM: light microscopy
rgb: red, green, blue image mapping
ssLM: serial section light microscopy
ssTEM: serial section transmission electron microscopy
TEM: transmission electron microscopy
