# Supplementary figures and images for: A Computational Framework for Ultrastructural Mapping of Neural Circuitry
Source: PLoS Biol. 2009 Mar 31;7(3):e1000074. doi: 10.1371/journal.pbio.1000074 (PMC2661966; doi:10.1371/journal.pbio.1000074)

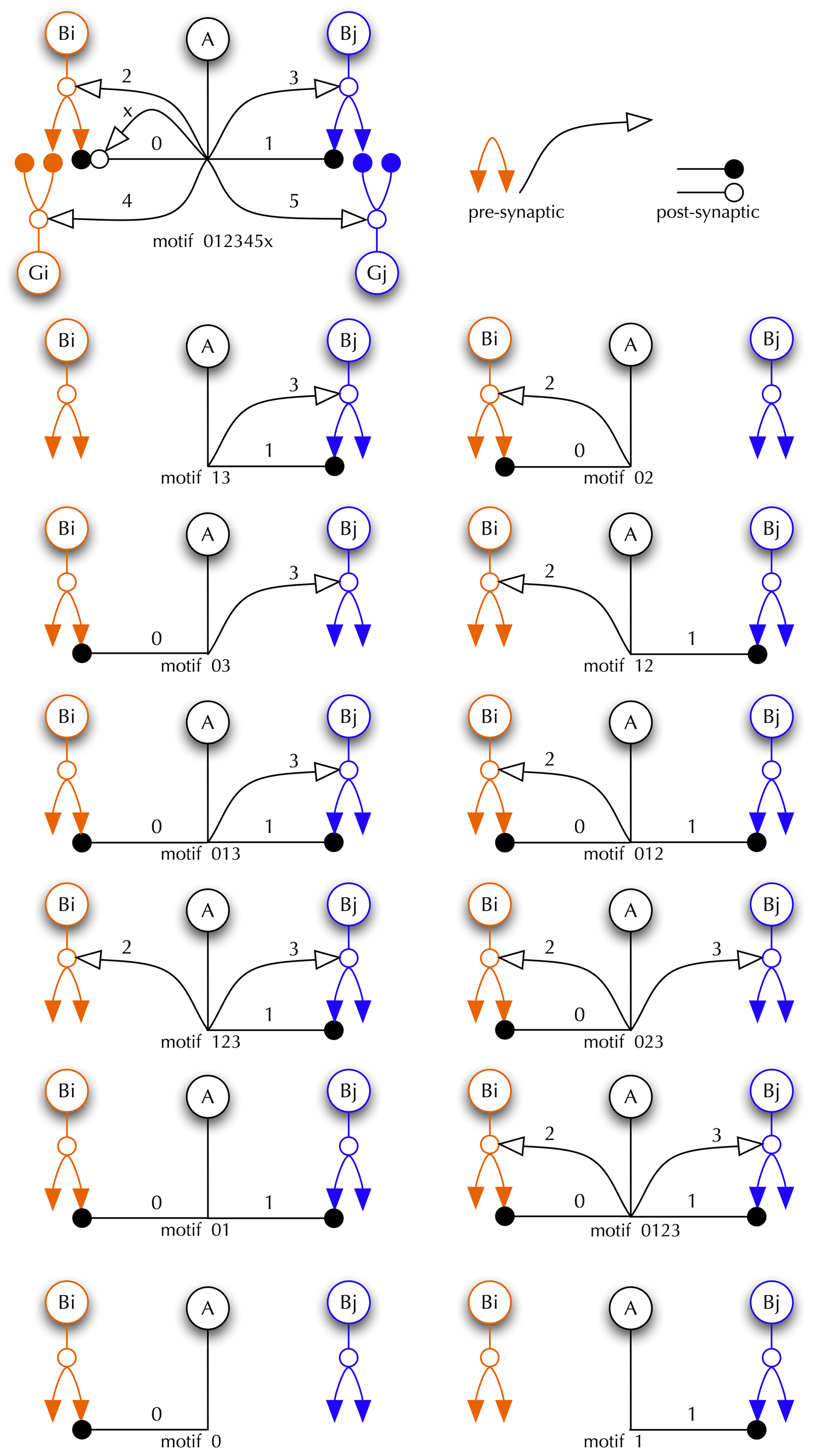

Supplement: Figure S1 — Assume that you have five kinds of cells: two different BCs (Bi, Bj), two different GCs (Gi, Gj), one AC (A) connecting them. The required vertical channels are Bi → Gi; Bj → Gj. The lateral channel options are: A → none: processes 0 and 1; A → Bi, Bj (feedback): processes 2 and 3; A → Gi, Gj (feedforward): processes 4 and 5; A → A (nested feedback): loop x. We start with a summary of submotifs. There are 12 allowed BCAC submotifs: motif → label; 0 → i mono input, no feedback; 1 → j mono input, no feedback; 01 → dual input, no feedback; 02 → i mono input, in-channel feedback; 03 → i mono input, cross-channel feedback; 12 → j mono input, cross-channel feedback; 13 → j mono input, in-channel feedback; 012 → dual input, feedback I; 013 → dual input, feedback j; 023 → i mono input, dual feedback; 123 → j mono input, dual feedback; 0123 → dual input, dual feedback. There are four possible AC → GC feedforward submotifs. A → none; A → Gi; A → Gj; A → Gi and Gj. There are two AC → AC nested feedback submotifs. A → none; A → A. The total combined motif number is ((12 BC → AC)(4 AC → GC) − 3)(2 AC → AC) = (45 basic submotifs)(2 nested forms) = 90. In connective terms this calculation includes −3 because motifs 0, 1, 01 cannot also have AC → no GCs. However, there are AC volume release mechanisms (peptides, NO, monoamines) that could have this motif. In a strict biological sense, there could be least 96 motifs. But in practice we might require constraints (see Figure S2). How many network motifs can you make with five or six retinal neurons? (1.29 MB TIF) [file pbio.1000074.sg001.tif]

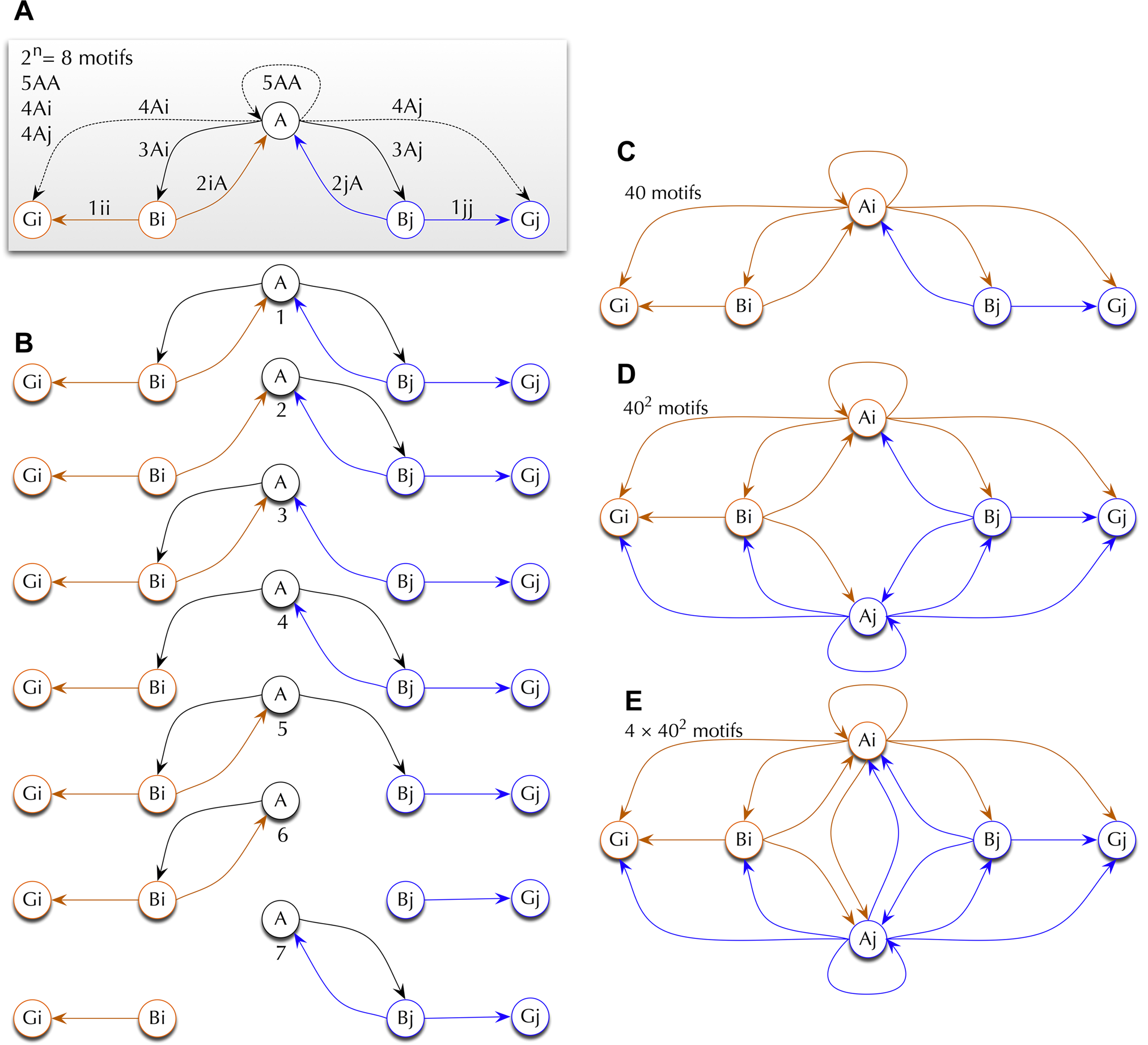

Supplement: Figure S2 — Even if we add the strong biological constraint that a minimal submotif must have a path to both GCs and one cross channel AC path (i.e., Bi → Ai → Bj), there are still many possible motifs. Here we redraw the pattern in Figure 1 to simplify the analysis. (A) This is the complete five-neuron network. Three connections (5AA, 4Ai, 4Aj) can be independently removed: 23 = 8 motifs are possible with these connections varied. (B) After removing 5AA, 4Ai, and 4Aj we have a basic submotif (1) that can be decimated by removing connection pairs. Submotifs 1–5 satisfy our constraint, thus admitting 40 biologically likely networks. Submotifs 6 and 7 are biologically plausible, but don't form a single network. What happens if we add one more AC? (C) This is the complete 40-motif network. (D) If one more AC is added (Aj), it represents an independent 40-motif network and the total possible paths becomes 402 = 1,600. (E) By adding connections between Ai and Aj increases the number 4-fold (none, Ai → Aj, Aj → Ai, Ai ↔ Aj) or 6,400. Thus the diversity of possible connections is so high that the most effective way to discover neural circuits is to actually map them by ssTEM. (KB TIF) [file pbio.1000074.sg002.tif]
