# Supplementary material for: A Computational Framework for Ultrastructural Mapping of Neural Circuitry
Source: PLoS Biol. 2009 Mar 31;7(3):e1000074. doi: 10.1371/journal.pbio.1000074 (PMC2661966; doi:10.1371/journal.pbio.1000074)
Supplement: Protocol S1 — (836 KB PDF) [file pbio.1000074.sd001.pdf]

A Computational Framework for Ultrastructural Mapping of Neural Circuitry Supplementary Protocols

J.R. Anderson[1], B.W. Jones[1], J-H Yang[1], M.V. Shaw[1], C.B. Watt[1], P. Koshevoy[2,3], J. Spaltenstein[3], E. Jurrus[3], Kannan U.V.[3], R. Whitaker[3], D. Mastronarde[4] , T. Tasdizen[3,5], R.E. Marc[1]

Affiliations: [1]Dept. Ophthalmology, Moran Eye Center, University of Utah; [2]Sorenson Media, Salt Lake City, UT; [3]Scientific Computing and Imaging Institute, University of Utah; [4]The Boulder Laboratory For 3-D Electron Microscopy of Cells, University of Colorado, Boulder; [5]Dept. Electrical and Computer Engineering, University of Utah.

Details of image processing tools

ir-fft

The purpose of *ir-fft* is to find the ordering of random image tiles. The first sub-problem is to find pairs of overlapping tiles. The main constraint at this stage of the algorithm is computational complexity because this procedure will be applied to approximately n^2 pairs where n is the total number of tiles in a section. If we restrict the class of allowed coordinate transformations between pairs of tiles to translation, a fast closed-form solution exists [1]. Let $F[S](u,v)$ denote the two-dimensional Fourier transform of image $S(x,y)$. For simplicity we refer to $F[S](u,v)$ as $F[S]$. The Fourier transform shift property [2] provides a simple rule relating the Fourier transforms of an image and a shifted version of it:

$F[S(x\text{-}xo,\ y\text{-}yo)] = e^{-j(uxo+vyo)} F[S(x,y)]$ (1)

Following Girod and Kuo [1], we define a displacement probability image between two images Si and Sj :

$Pi,j\ (x,y) = Real\ [F^{-1}\ [\ \Phi ij\ (\Phi ii\ \Phi jj)^{-1/2}]$ (2)

where $\Phi ij = F[Si\ ]\ F[Sj\ ]^*$ is the cross-correlation of the two images, $F[Sj]^*$ being the complex conjugate. Similarly, Φii and Φjj are the auto-correlations. Using the Fourier transform shift property defined in Equation (1) it can be shown that if Si and Sj differ only by a displacement vector (xo,yo), the displacement probability image in Equation (2) reduces to a Dirac function

$Pij\ (x,y) = \delta\ (x\text{-}xo,y\text{-}yo)$,

assuming periodicity of the images. In other words, $Pij\ (x,y)$ will have a non-zero entry for only a single displacement vector. For partially overlapping images, the exact relationship $Pij\ (x,y) = \delta(x\text{-}xo,y\text{-}yo)$ does not hold. However, if the amount of overlap is sufficient, the maximum of $Pij\ (x,y)$ should correspond to the true displacement between images Si and Sj. In practice, finding this maximum is non-trivial because for most TEM images P is very noisy. Also, Pij for two non-overlapping images may contain several weak maxima, or none at all. These problems were not addressed in Girod and Kuo [1].

We have found four steps necessary to identify the location of the correct maxima in P. The first is to pre-smooth images to reduce noise. The second is to select and apply a threshold to P to isolate global peaks. We choose the threshold at the 99th percentile of the histogram of P: i.e. 1% of the total pixels in P are considered as possible maxima locations. Third, we locate clusters of at least five 8-connected pixels, indicating a strong maximum. If the maxima are scattered across P, it is likely there is no strong maximum. Maxima coordinates are calculated as the centers of mass of the corresponding clusters. The final step is to verify which, if any, of these maxima is the true displacement between the image pair. Non-overlapping image pairs typically produce a Pij with several maxima points at roughly the same value, while the Pij of two matching tiles produces one maximum significantly stronger than the rest. If this maximum is at least twice as strong as all others, it is marked as a good match; otherwise, we conclude that the tiles do not overlap. Displacement vectors with less than 5% overlap are discarded since true tiles are expected to have as much as 15% overlap. The method works best for tiles with at least 10% overlap. For further details, we refer the reader to Koshevoy et al. [3].

The second sub-problem is finding the correct layout of the tiles into the mosaic space. For any pair of tiles, alternative mappings exist. A direct mapping exists if the pair was determined to overlap. But we also track cascaded mappings via intermediate tiles. For example, there may exist a mapping $S0 : S1$ between tiles $S0$ and $S1$, and another mapping $S1 : S4$ between tiles $S1 : S4$. A mapping $S0 : S4$ between tiles $S0$ and $S4$ can be created via the intermediate tile $S1$ as an alternative to the direct

mapping. The mapping with the least cost is preferred even when it has greater cascade length. The cost function is the sum of squared intensity differences in the overlap region, normalized by the overlap area. Using these redundant mappings between any two tiles presents a great opportunity to select the best mapping possible, especially if an error occurred in the computation of the direct mapping. To build a mosaic, we select an arbitrary tile as an anchor or first tile. Then the tile with the lowest best mapping cost to the anchor image, computed as described above, is placed into the mosaic. Tiles are successively placed always choosing the best mapping to tiles already in the mosaic.

It is possible that meso-scale brightness variations might compromise mosaic alignments. Tiles with problematic intensity variations can be preprocessed using *ir-clahe* (Contrast Limited Adaptive Histogram Equalization; CLAHE) before auto-tiling with *ir-fft*, and/or with *ir-blob* (a coarse scale blob and edge detector) prior to registering assembled slices with *ir-stos-brute* (see below). Depending on the user's preferences, the computed transforms can be applied to either the pre- or unprocessed tiles for visualization with *ir-assemble*, *ir-stom* (slice-to-movie) or the other viewers.

ir-translate

Using the approximate positions from metadata, only tiles that are known to overlap are matched using the Fourier shift method previously described. This reduces the complexity of the method from a quadratic to a linear function of the number of tiles. Next, a tension vector proportional to the offset between the approximate position, and the preferred position as found by matching, is defined. This tension vector can be thought of as a spring between the two tiles pulling the two tiles toward their preferred relative position. Most tiles will have multiple neighbors and will therefore experience multiple tensions. The sum of the tension vectors experienced by each tile will be a net force pushing each tile towards a more desirable position. Continuing the analogy of multiple springs, the energy of each tension vector can be thought of as the potential energy stored in a spring, thus a global system energy can be calculated by computing the sum of the squares of all the tension vectors. The algorithm iterates over each tile in the slice multiple times, nudging all the tiles with each pass. This will result in finding a local minimum of the global system energy.

ir-refine-grid

Non-linear warp refinement and is accomplished with *ir-refine-grid*. During the earlier stages of algorithm development, several continuous polynomial transforms were explored, in particular a bivariate cubic radial distortion transform and a bivariate cubic Legendre polynomial transform. These transforms suffer from a trade-off where the stability of the transform is related inversely to the degree of the polynomial. Our final approach uses a discontinuous transform with local phase correlation. Each tile is sampled onto a coarse uniform triangle mesh. Each vertex in the mesh stores two sets of coordinates: the local tile coordinates and the mosaic space coordinates. The image is warped by changing the mosaic space coordinates directly. To map a coordinate from the mosaic space into the tile space, the tile mesh is searched for the triangle containing the given mosaic space point. Then, the barycentric coordinates of the intersection point are used to calculate the corresponding tile space point by interpolating the tile space vertex coordinates. The mapping from tile space into mosaic space is trivial due to the uniform structure of the triangle mesh in the tile space. One has to find the mesh quad containing the tile space point and perform a bilinear interpolation between the mosaic space coordinates of the quad vertices. These operations are implemented in an efficient manner using *OpenGL* (Open Graphics Library).

At each vertex in the mesh a small, corresponding neighborhood is sampled from all of the tile neighbors in the mosaic. The neighborhood need only be large enough to capture a meaningful amount of image texture for phase correlation to work: 96x96 pixels. The mesh nodes are spaced at approximately one third of the neighborhood size. The two neighborhoods are matched using the same Fourier shift method described for *ir-fft*. The dense field of displacement vectors produced by this matching is used to correct the mosaic space coordinates of the vertex. It is possible for tile matching to produce mismatches, so the calculated vectors at each vertex are median filtered to remove the outliers. The vectors are further de-noised with a Gaussian smoothing filter. This post-processing requires 2-3 passes to ensure convergence. For further details, we refer the reader to Koshevoy [4].

ir-tweak

ir-tweak is a manual image registration tool using thin-plate spline transforms implemented using OpenGL, Qt (the “Cute” Widget Toolkit), ITK (The Insight Segmentation and Registration Toolkit), GLEW (OpenGL Extension Wrangler Library), and Cg (C for Graphics shading library developed by Nvidia and Microsoft). *Ir-tweak* implements fragment shading, reducing the memory footprint of the image textures and uses a thin-plate spline transform.

A Tutorial on Computational Molecular Phenotyping (CMP)

Concept

CMP is based on four technologies:

- quantitative immunoprobes
- ultrathin section arrays
- imaging
- computational N-space classification

CMP transforms complex image sets that must otherwise be qualitatively perused by humans (>10^2 sections, > 10^6 cells, 8-36 probes) into N-space data matrices viewed by an algorithmic operator – a statistical pattern classifier [5]. The classifier parses the matrices and allows a reverse transform into a theme map optimized for human visualization. Theme maps index the underlying N-space molecular signatures as well as structural signatures (cell size, position, density, texture, patterning, etc). A molecular+structural signature is the quantitative realization of a phenotype. It is continuously extensible and managed by well-known statistical rules. A detailed description of CMP calibration, imaging, visualization and clustering is provided in the appendix to [6] Marc et al (1995) Pattern recognition of amino acid signatures in retinal neurons. J Neurosci 15: 5106-5129. This is an abbreviated overview of some of that content.

History

The notion of a characteristic molecular signature was developed independently by Thomas Hökfelt and Dominic Lam in the mid-1980's (e.g.[7]). The practical concept of characterizing cells by small molecule signatures was developed by Ottersen and his colleagues [8,9,10,11]. The Marc laboratory began developing optical CMP using silver-intensified immunogold methods in 1986, some four years after code for analysis of multispectral satellite imagery was declassified and commercialized (see PCI Geomatica Link). The data structures and interpretive objectives of immunocytochemical analyses are congruent to remote sensing structures and goals [6,12]and are based on classification theory and Bayesian statistics. Studying classes of structures involves two questions.

Traditional parametric statistics asks: Are x and y drawn from the same distribution?

Classification asks a more challenging question: Did x come from distribution A or B or C …?

This challenge is addressed in the publications of Fix and Hodges [13,14], Cover and Hart [15], Tou and Gonzales [16], Duda & Hart [17]. These are the "Hodgkin-Huxley" papers of classification theory.

Rationale

CMP approaches the ideal classification method for heterocellular metazoan tissues. There are two antipodal designs for classifying cells: Univariate and multivariate classification. The univariate design is the standard immunocytochemical approach. It requires one validated probe per class and hundreds of samples to test them all. Validation is tissue and species specific. For example, the retina clearly has more than >60 cell classes based on several estimates and tracking them all would require

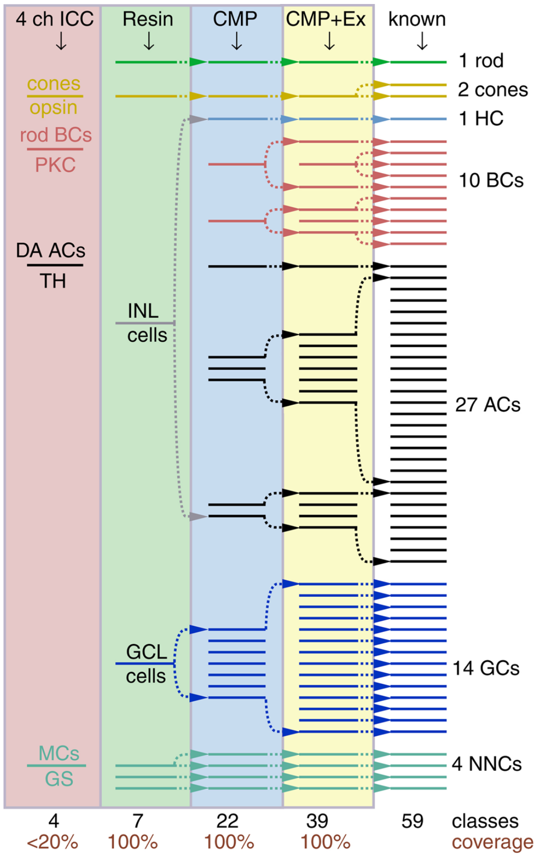

Figure CMP 1. Histograms of classification comparing four-channel immunocytochemistry (red column), resin microscopy (green column), CMP (blue column), and CMP + excitation mapping (yellow column) over the known class range of the mammalian retina (white column). Each horizontal band represents a class or superclass. The numbers of bands in a column indicate a method's absolute resolution. CMP expands resin classes into 22 unique superclasses and CMP + excitation mapping nearly doubles that. Only CMP provides strong classification and 100% coverage. "4 ch icc" uses cone opsin, PKC α, TH (tyrosine hydroxylase) and GC (glutamine synthetase) as markers. NNCs , non-neuronal cells.

>60 validated probes. An example of the best univariate work is Haverkamp and Wässle [18], and they specifically note: "Only a few markers labeled only one cell type: Most antibodies recognized specific groups of neurons".

The weaknesses of the univariate approach are:
- most essential probes don't yet exist or haven't been validated
- species-independence cannot be assured
- data fusion is usually impossible
- no proof of completeness or correctness is possible

Conversely, multivariate classifiers use *basis sets* (linearly independent measures in a vector space) of probes targeting overlapping classes to create an N-space matrix for a single sample. The strengths of multivariate CMP approach are:
- probes for concurrent use exist
- all small molecule probes are species independent and work in all tissues, in all organisms
- data fusion methods exist and are robust
- mathematically complete coverage is assured

Fig. CMP 1 graphically compares the classification power of univariate immunocytochemistry with CMP in the mammalian retina. Even a theoretically optimal conventional preparation on one frozen section with 4 channels of immunocytochemical data of captures but a tiny segment of the known classes with qualitative signals and poor coverage. Conversely CMP and CMP + excitation mapping [19,20,21] successively expand class resolution to high levels with 100% cell coverage. Every cell gets tagged with an N-space signature, even if the biological meaning is yet unknown. The multivariate CMP approach is also extensible as selected macromolecular [22,23] and ultrastructural signals [24,25] can be included. CMP packs data to unprecedented densities. CMP analysis is independent of section thickness [6], and ultrathin sectioning allows high sampling densities in every cell. A single 10 µm wide cell yields = 250 ultrathin 40 nm sections. We can compress up to 12-36 channels of quantitative data into twelve such serial sections, using less than 5% of the cell.

Probe Libraries: Theory & Practice

CMP uses multiple basis probes to increase N-space resolution and coverage. CMP probes can track intracellular concentration differences as small as 40 µM [21] (Fig. CMP 2). This gives robust classifying power. We think of discrete 2D image metrics as pixels, 3D volume metrics as voxels, and N-space metrics as "nixels". The resolution R of a classification space is $R = \prod \Delta S_n$ where n is a molecular channel, ΔS is signal resolution, and the

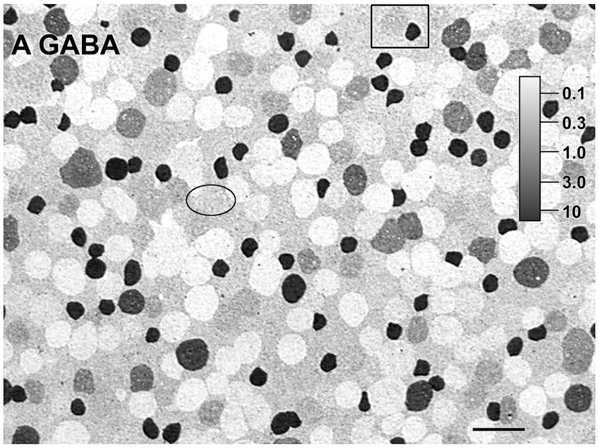

Figure CMP 2. Differential GABA content of cells in the ganglion cell layer of the rabbit retina visualized by quantitative GABA mapping on a 250 nm section. Scaling was determined from artificial standards. Cellular contents range from 50 µM to 10 mM. Some ganglion cells are literally invisible in the array because they have the same GABA content as the surrounding Müller cells (ellipse). Other cells can be differentiated from the Müller cells by having contents roughly 40 µM higher (box); a strongly immunoreactive starburst amacrine cell is also contained in the box. The image is density scaled. Scale bar, 25 µm. From Marc and Jones [21].

power function is evaluated over the interval n=1 to N, the total number of channels. With N=8 channels, a minimum ΔS=8 resolved steps per channel (3 bits, much less than achieved), $R = 16.8 \times 10^6$ nixels of molecular resolution for segmenting retina into 60 hypervolumes of $> 2 \times 10^5$ nixels/class. Thus CMP has intrinsic classification power.

In 1945 Karl Landsteiner [26] demonstrated regio- and stereospecific binding of small molecules by IgGs and tissue level discrimination of even enantiomers (e.g. D- and L- aspartate) is now routine [6], as is targeting free amines in general [6,8,12,21,27,28,29,30,31,32,33,34]. Anti-hapten IgGs have successfully been made against targets as complex as C60 fullerenes, as nonpolar as cholesterol, or as large as vitamin B12 [35,36,37]. Most small molecules are potential haptens. A large library of probes targeting small molecules is currently in use in the Marc laboratory. See CMP Probes Link.

Quantitation

IgG binding in resin-based CMP is limited to the surfaces of sections [6], making CMP a 2-phase assay with better physical properties for quantitation than traditional immunocytochemistry [12,21]. CMP can be calibrated against standards containing known amounts of target hapten (e.g. Marc and Jones, [21]). Either fluorescence (intensity) or density methods may be used. CMP based

on silver-intensified immunogold [38], has advantages of high sensitivity, good dynamic range and archival imaging. Fig. CMP 2 is reproduced from Marc and Jones [21] and demonstrates both the range and precision of CMP, calibrated directly against artificial standards. Marc et al. [12] also showed direct calibration using immunodot assays. The practical sensitivity of CMP ranges from 50 uM to 10 mM, which covers the range of differential expression in most cells. By using standardized image capture protocols [21], CMP imagery is quantitative and allows direct readout of cellular concentrations according to the "Silver" equation: $P = (C^n \cdot Pmax)/(C^n + \sigma^n)$, where P = pixel value, Pmax is the saturated pixel value, C is concentration, n is the cooperativity (analogous to video gamma) and σ is the concentration at P=0.5 (Fig. CMP 3). However, transforming images to concentration maps is not necessary for quantitative statistical comparisons, segmentation, or classification.

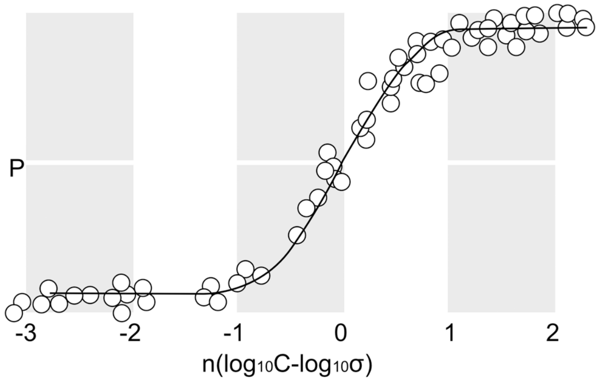

Figure CMP 3. Calibrating CMP. Competitive displacement curves for IgG D, IgG E, IgG J, IgG Q, IgG γ run concurrently and aligned at σ, the half saturation point of the Silver Equation. The curves were pinned by visualizing IgG E binding to an artificial standard stack.

Ultrathin section arrays

The second key to CMP is production of high-quality section arrays. A detailed protocol is located at CMP Protocol Link. There are three elements to the production of high quality arrays: fixation, resin embedding, and sectioning.

Fixation. The detection regime for small amines is a uses glutaraldehyde (GA) cross-linking (e.g. Marc and Liu, [24]). This forms a dense matrix with an average pore diameter of 2-5 nm. IgGs (≈ 150 kD) cannot penetrate the matrix. Thus the precise surfaces produced by resin ultramicrotomy are ideal and independent of section thickness (Fig. CMP. 4). Immunogold detection using section etching is quantitative and reproducible [6,9,12]. The epitope surface 'above' the matrix is likely to be only ≈ 1 nm deep and, in this volume, a 10 mM target thus exposes ≈ 6000 randomly oriented epitopes/μm². Our routine detection limit with silver-intensification is ≈ 50 μM, corresponding to 30 true targets/μm². These and other calculations suggest that the efficiency of small molecule trapping is very high, perhaps 85% or higher (see CMP Substrates Link)

Resin embedding and sectioning. We have tested many resin options and none approaches the flexibility and stability of epoxide resins with dodecenylsuccinic anhydride cross-linking and 2,4,6 tris-(dimethyaminomethyl)-phenol end-linking. This resin is electron-beam stable

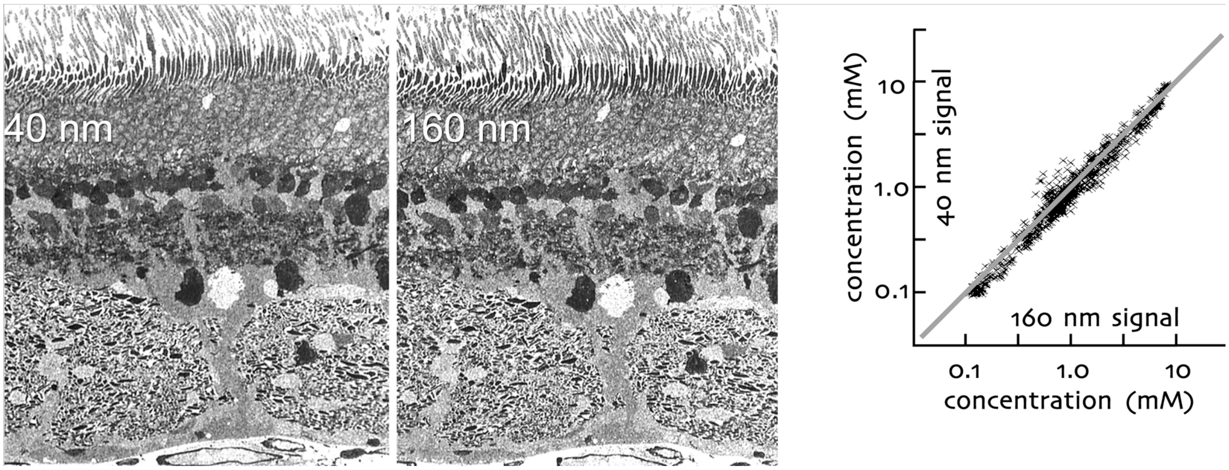

Figure CMP 4. CMP precision. Ultrathin resin sections of rabbit retina probed for glutamate and visualized with calibrated silver metallized immunogold binding. All signal in the images is glutamate detection. There is no 'background' and the SNR > 10^3:1. The left section is 40 nm thick and the right 160 nm. The scatterplot demonstrates that the signals are identical and correlated over the entire metallization range.

and allows ultrathin sectioning, but remains fully removable by anhydrous methoxide etching. Routine CMP is done with 200 nm diamond-cut serial sections floated onto 25 µl ultrapure water droplets on ultraclean array spot slides (HTC® hydrophobic mask slides, Thermo Scientific). Sectioning is done manually and any qualified ultrastructural technician can readily prepare section arrays. Resin embedding also allows multiplexing of samples [12,19] and as many as a dozen tissues can be reformatted into a single bloc (see CMP Plastic Protocols). Figure CMP 5 shows a typical 12-well tissue array.

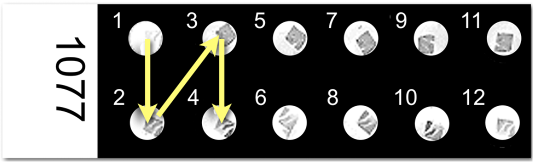

Figure CMP 5. A typical 12-well tissue array with sections positioned in a repeating reverse N pattern, visualized with silver metallization.

Imaging

Because silvered CMP is completely archival, images can be captured under optimal conditions of signal-to-noise, with stabilized power supplies and any good 8-bit scientific monochrome camera. We prefer capturing entire sections at a resolution of 183 nm/pixel (slightly oversampled) with a 1.0 NA planapochromatic oil immersion objective and high-resolution scanning stage. Capture and montaging protocols are detailed in Marc and Cameron [39] and Marc and Jones [21] and at the CMP Protocol Link. Captured images are mosaicked and registered into large multispectral datasets for classification.

Classification

Pattern recognition theory was developed in large part for multispectral analysis of planetary imaging data. A set of serial sections probed for small molecules is similar to a series of planetary photographs taken through different spectral filters: common loci across images will index a list of values representing planetary spectral reflectance or small molecule contents. Viewing the data as color triplets aids in finding the obvious relations, but is not a statistical assessment. The more powerful is pattern recognition in N-space, and clustering in particular. Algorithms such K-means and isodata clustering extract N-dimensional means and variances for classes by calculating hypersurface decision boundaries [16,17]. How such algorithms work is explained in great detail in Marc et al. ([6]).

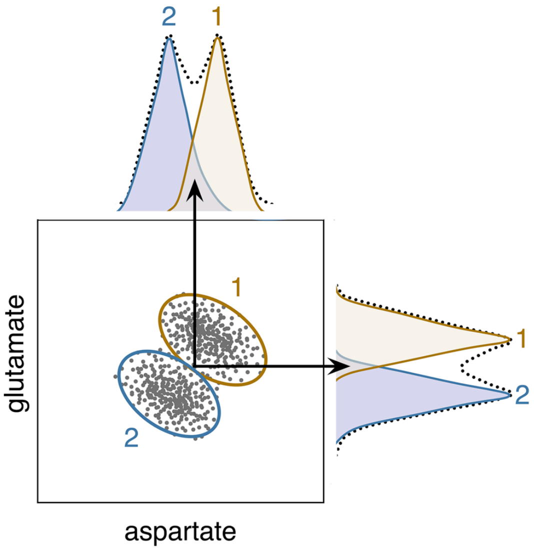

Figure CMP 6. Summary bivariate distributions of idealized glutamate and aspartate signals for two model cell classes 1 and 2. The ellipses are the 2 SD borders of the bivariate data sets and the unimodal profiles are the marginal distributions for each cell class. A datapoint in class 2 possessing an indeterminate signal in univariate space is indicated by arrows. Revised from Marc et al., 1995.

Fig. CMP 6 is an example of data from cells with overlapping distributions in univariate space though separable in 2-space. Visually interpreting data sets of higher dimensionality is difficult or impossible, but computing classes and decision boundaries is not. Thus a map of theme class memberships can be generated [6,21,39] and explored to visualize the underlying signatures in more detail. Derived classes can be tested for their statistical separabilities by a probability of error (p_e) assessment, typically by calculating the N-space distances of the data clouds (e.g. transformed divergence or Bhattacharyara distance) and modeling p_e. This is a powerful measure, because separable classes are always significant, but not *vice versa*. Though the bivariate distributions have little overlap and a low p_e, the univariate data overlap extensively with high p_e since univariate data cannot encode covariance. If one were required to name the group from which a given sample arose based on the strength of one type of signal alone, the error would be unacceptably high. For example, the gray cell indicated at the top of Figure CMP 5 would be misclassified 50% of the time and the integrated error for the entire range of data would be 0.2, over an order of magnitude higher than the bivariate p_e. Such univariate errors are nontrivial and common in immunocytochemistry.

The results of K-means classifications can be visualized many ways:

- As theme maps (i.e. political maps) by color coding every pixel in the original image according to theme class ([21]; CMPView © JA Anderson; Fig. CMP 7)
- As bivariate probability density distributions (Fig. CMP 6)
- As matrices of univariate probability density distributions viewed as a small multiple plot ([40]; CMPView © JA Anderson)
- As 2N-plots where each cell is mapped as a set of superimposed bivariate plots ([21]; CellKit © RE Marc; Fig. CMP 8)
- As parallel plots (CMPView © JA Anderson) developed by Inselberg [41], on which 2N-plots were based.

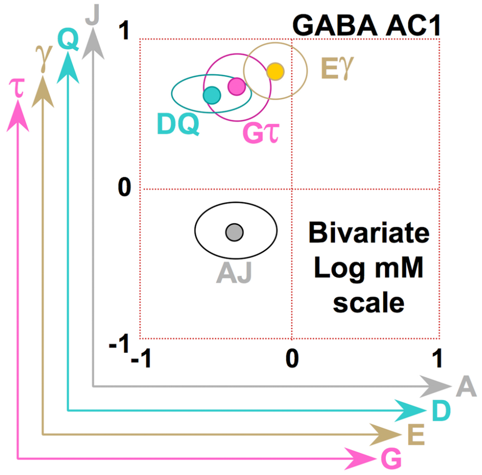

Figure CMP 8. N-plot for the ADEGJQτγ basis set. This N-plot was derived from the molecular phenotype data of zebrafish class 1 GABA amacrine cells (GABA AC1). Each dot encodes a bivariate mean on a logarithmic mM scale (i.e. –1 = 0.1 mM, 0 = 1 mM, 1 = 10 mM), and is bounded by a 1 standard deviation ellipse. Four pairs of points represent a projection of 8-space into 2-space, and distinctions among classes are thus represented by the differing patterns of signals in N-plots (see Marc and Jones, 2001). The XY pairs are arbitrarily formed but constant throughout this manuscript: AJ (grey), DQ (cyan), Eγ (gold), Gτ (magenta). In this plot, it can be quickly determined that the mean A, D, G, E and J values are less than 1 mM in this class, because their coordinates are all below the log 0 mM line. Conversely, the mean Q, γ and τ values are all >> 1 mM.

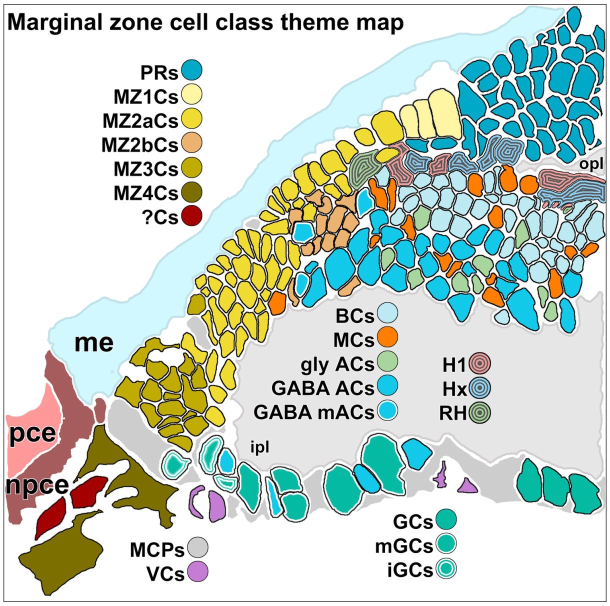

Figure CMP 7. A formal theme map of the zebrafish retinal marginal zone. Each cell type is coded by class membership based on isodata clustering and histogram deconvolution. Abbreviations: iGCs, immature ganglion cells; MCPs, Müller cell processes; mGCs, maturing ganglion cells; GABA mACs, maturing GABA amacrine cells; MZ1Cs, marginal zone 1 cells; MZ2aCs, marginal zone 2a cells; MZ2bCs, marginal zone 2b cells; MZ3Cs, marginal zone 3 cells; MZ4Cs, marginal zone 4 cells, PRs, photoreceptors; ?Cs, unknown cells associated with MZ4; VCs, vascular cells. From Marc and Cameron, 2003.

Supplementary Material References

1. Girod B, Kuo D (1989) Direct estimation of displacement histograms. Proceedings of the Optical Society of America Meeting on Understanding and Machine Vision: 73-76.

2. Gonzalez RC, Woods RE (2008) Digital Image Processing: Pearson Prentice Hall.

3. Koshevoy PA, Tasdizen T, Whitaker R, Jones BW, Marc RE (2006) Assembly of large three-dimensional volumes from serial section transmission electron microscopy. . Proceedings of 2006 MICCAI Workshop on Microscopic Image Analysis with Applications in Biology: 1-8.

4. Koshevoy PA, Tasdizen T, Whitaker R (2007) Automatic assembly of TEM mosaics and mosaic stacks using phase correlation. Salt Lake City: University of Utah.

5. Duda RO, Hart PE, Stork DG (2000) Pattern Classification. NY: Wiley-Interscience. 654 p.

6. Marc RE, Murry RF, Basinger SF (1995) Pattern recognition of amino acid signatures in retinal neurons. J Neurosci 15: 5106-5129.

7. Lam DM, Li HB, Su YY, Watt CB (1985) The signature hypothesis: co-localizations of neuroactive substances as anatomical probes for circuitry analyses. Vision Res 25: 1353-1364.

8. Ottersen OP, Stormmathisen J (1984) Glutamate-Containing and Gaba-Containing Neurons in the Mouse and Rat-Brain, as Demonstrated with

a New Immunocytochemical Technique. Journal of Comparative Neurology 229: 374-392.

9. Ottersen OP (1987) Postembedding light- and electron microscopic immunocytochemistry of amino acids: description of a new model system allowing identical conditions for specificity testing and tissue processing. Exp Brain Res 69: 167-174.

10. Ottersen OP (1989) Postembedding Immunogold Labeling of Fixed Glutamate - an Electron-Microscopic Analysis of the Relationship between Gold Particle Density and Antigen Concentration. Journal of Chemical Neuroanatomy 2: 57-66.

11. Ottersen OP, Zhang N, Walberg F (1992) Metabolic Compartmentation of Glutamate and Glutamine - Morphological Evidence Obtained by Quantitative Immunocytochemistry in Rat Cerebellum. Neuroscience 46: 519-534.

12. Marc RE, Liu WL, Kalloniatis M, Raiguel SF, van Haesendonck E (1990) Patterns of glutamate immunoreactivity in the goldfish retina. J Neurosci 10: 4006-4034.

13. Fix E, Hodges LJL (1951) Discriminatory analysis: Nonparametric discrimination: Consistency properties. Randolph Field, TX: USAF school of Aviation Medicine. 261-279 p.

14. Fix E, Hodges LJL (1952) Discriminatory analysis: Nonparametric discrimination: Small sample performance. Randolph Field, TX: USAF school of Aviation Medicine. 280-322 p.

15. Cover TM, Hart PE (1967) Nearest neighbor pattern classification. IEEE Transactions on Information Theory IT-13: 21-27.

16. Tou JT, Gonzales RC (1974) Pattern Recognition Principles. New York: Addison-Wesley.

17. Duda RO, Hart PE (1973) Pattern Classification. NY: Wiley-Interscience.

18. Haverkamp S, Wässle H (2000) Immunocytochemical analysis of the mouse retina. J Comp Neurol 424: 1-23.

19. Marc RE (1999) Mapping glutamatergic drive in the vertebrate retina with a channel-permeant organic cation. J Comp Neurol 407: 47-64.

20. Marc RE (1999) Kainate activation of horizontal, bipolar, amacrine, and ganglion cells in the rabbit retina. J Comp Neurol 407: 65-76.

21. Marc RE, Jones BW (2002) Molecular phenotyping of retinal ganglion cells. J Neurosci 22: 413-427.

22. Marc RE, Jones BW, Anderson JR, Kinard K, Marshak DW, et al. (2007) Neural reprogramming in retinal degeneration. Invest Ophthal Vis Sci 48: 3364-3371.

23. Marc RE, Jones BW, Watt CB, Vazquez-Chona F, Vaughan DK, et al. (2008) Extreme retinal remodeling triggered by light damage: implications for age related macular degeneration. Molecular Vision 14: 782-806.

24. Marc RE, Liu W (2000) Fundamental GABAergic amacrine cell circuitries in the retina: nested feedback, concatenated inhibition, and axosomatic synapses. J Comp Neurol 425: 560-582.

25. Jones BW, Watt CB, Frederick JM, Baehr W, Chen CK, et al. (2003) Retinal remodeling triggered by photoreceptor degenerations. Journal of Comparative Neurology 464: 1-16.

26. Landsteiner K (1945) The specificity of serological reactions. Boston: Harvard University Press. 450 p.

27. Storm-Mathisen J, Leknes AK, Bore AT, Vaaland JL, Edminson P, et al. (1983) 1st Visualization of Glutamate and Gaba in Neurons by Immunocytochemistry. Nature 301: 517-520.

28. Geffard M, Segula P, Heinrich-Rock AM (1984) Antisera against catecholamines: specificity studies and physicochemical data for anti-dopamine and anti-p-tyramine antibodies. Molecular Immunology 21: 515-522.

29. Steinbusch HWM, Wouterlood FG, deVente J, Bol JGJM, Berkenbosch F (1988) Immunohistochemical localization of monoamines and cyclic nucleotides. Acta Histochem S35: 85-106.

30. Stormmathisen J, Ottersen OP (1990) Immunocytochemistry of Glutamate at the Synaptic Level. Journal of Histochemistry & Cytochemistry 38: 1038-1038.

31. Pow DV (1993) Immunocytochemistry of Amino-Acids in the Rodent Pituitary Using Extremely Specific, Very High-Titer Antisera. Journal of Neuroendocrinology 5: 349-356.

32. Pow DV, Crook DK (1993) Extremely High-Titer Polyclonal Antisera against Small Neurotransmitter Molecules - Rapid Production, Characterization and Use in Light-Microscopic and Electron-Microscopic Immunocytochemistry. Journal of Neuroscience Methods 48: 51-63.

33. Kalloniatis M, Marc RE, Murry RF (1996) Amino acid signatures in the primate retina. J Neurosci 16: 6807-6829.

34. Marc RE, Murry RF, Fisher SK, Linberg KA, Lewis GP (1998) Amino acid signatures in the detached cat retina. Invest Ophthal Vis Sci 39: 1694-1702.

35. Izhaky D, Pecht I (1998) What else can the immun system recognize? Proc Natl Acad Sci 95: 11509-11510.

36. Chen BX, Wilson SR, Das M, Coughlin DJ, Erlanger BF (1998) Immunology Antigenicity of fullerenes: Antibodies specific for fullerenes and their characteristics. Proc Natl Acad Sci 95: 10809-10813.

37. Birn H, Willnow TE, Nielsen R, Norden AGW, Bönsch C, et al. (2002) Megalin is essential for renal proximal tubule reabsorption and accumulation of transcobalamin-B12. Am J Physiol Renal Physiol 282: F408-F416.

38. Kalloniatis M, Fletcher EL (1993) Immunocytochemical localization of the amino acid neurotransmitters in the chicken retina. J Comp Neurol 336: 174-193.

39. Marc RE, Cameron DA (2002) A molecular phenotype atlas of the zebrafish retina. J Neurocytol 30: 593-654.

40. Tufte ER (2001) The Visual Display of Quantitative Information: Graphics Press USA. 197 p.

41. Inselberg A, Dimsdale B. Parallel coordinates: a tool for visualizing multi-dimensional geometry; 1990; San Francisco CA. pp. 361–375.
